# Higgs pair production in vector-boson fusion at the LHC and beyond

**DOI:** 10.1140/epjc/s10052-017-5037-9

**Published:** 2017-07-19

**Authors:** Fady Bishara, Roberto Contino, Juan Rojo

**Affiliations:** 1 Rudolf Peierls Centre for Theoretical Physics, University of Oxford, 1 Keble Road, Oxford, UK; 2 Scuola Normale Superiore, Pisa and INFN Pisa, Pisa, Italy; 3 Institut de Théorie des Phénomenes Physiques, EPFL, Lausanne, Switzerland; 4 Theoretical Physics Department, CERN, Geneva, Switzerland; 5 Department of Physics and Astronomy, VU University Amsterdam, De Boelelaan 1081, 1081 HV Amsterdam, The Netherlands; 6 Nikhef, Science Park 105, 1098 XG Amsterdam, The Netherlands

## Abstract

The production of pairs of Higgs bosons at hadron colliders provides unique information on the Higgs sector and on the mechanism underlying electroweak symmetry breaking (EWSB). Most studies have concentrated on the gluon-fusion production mode which has the largest cross section. However, despite its small production rate, the vector-boson fusion channel can also be relevant since even small modifications of the Higgs couplings to vector bosons induce a striking increase of the cross section as a function of the invariant mass of the Higgs boson pair. In this work we exploit this unique signature to propose a strategy to extract the *hhVV* quartic coupling and provide model-independent constraints on theories where EWSB is driven by new strong interactions. We take advantage of the higher signal yield of the $b\bar{b} b\bar{b}$ final state and make extensive use of jet-substructure techniques to reconstruct signal events with a boosted topology, characteristic of large partonic energies, where each Higgs boson decays to a single collimated jet. Our results demonstrate that the *hhVV* coupling can be measured with 45% (20%) precision at the LHC for $\mathscr {L}=300$ (3000) fb$^{-1}$, while a 1% precision can be achieved at a 100 TeV collider.

## Introduction

Following the discovery of the Higgs boson in 2012 [1, 2], the measurement of its couplings to the other standard model (SM) particles has become one of the main goals of the LHC programme. In this respect double Higgs production provides a unique handle, in particular since it allows the extraction of the trilinear Higgs self-coupling $\lambda$. In addition to constraining $\lambda$, in the vector-boson fusion (VBF) channel double Higgs production also probes the strength of the Higgs non-linear interactions with vector bosons at high energies. This process can thus help establish the nature of the Higgs boson, whether it is a composite or elementary state or whether or not it emerges as a Nambu–Goldstone boson (NGB) of some new dynamics at the TeV scale [3–5].

Many scenarios of new physics beyond the SM (BSM) generically predict enhanced cross sections for Higgs pair production with or without the resonant production of new intermediate states; see for example Refs. [6–20]. For this reason, searches for Higgs pair production at the LHC by ATLAS and CMS have already started at 8 TeV [21–25] and $13\,$TeV [26–29], and will continue during Runs II and III, as well as at the high-luminosity LHC (HL-LHC) upgrade with $3\,\text {ab}^{-1}$ of integrated luminosity. On the other hand, in the SM the small production rates make a measurement of Higgs pair production extremely challenging even at the HL-LHC, and the ultimate accuracy could only be achieved at a future $100\,$TeV hadron collider [17, 30–34].

Similarly to single Higgs production, the dominant mechanism for Higgs pair production is the gluon-fusion mode [35]. This channel has been extensively studied in the literature and several final states have been considered, including $b\bar{b}\gamma \gamma$, $b\bar{b}\tau ^+\tau ^-$, $b\bar{b}W^+W^-$ and $b\bar{b}b\bar{b}$ (for a list of feasibility studies, see for example Refs. [17, 30, 36–43]). Working in the infinite top mass approximation, the gluon-fusion di-Higgs production cross section was calculated at NLO in [44] and NNLO in [45]. The resummation of soft-gluon emissions was performed at NNLL in [46, 47]. Beyond the $m_t\rightarrow \infty$ limit, the impact of top-quark mass effects on NLO QCD corrections was first determined in [48] through a reweighting technique based on an approximate two-loop matrix element and by [49, 50] in a $1/m_t$ expansion. Recently, the full NLO calculation was performed by [51]. Matching the fixed order computations to a parton shower was done at LO in [52] and at NLO in [53].

Recent studies indicate that Higgs pair production in gluon fusion at the HL-LHC will allow the extraction of the Higgs self-coupling $\lambda$ with $\mathscr {O}(1)$ accuracy, with details varying with the analysis and the specific final state; see Refs. [54–58] for the latest ATLAS and CMS estimates, as well as [17, 30, 38, 59, 60].

Higgs pairs can also be produced in the VBF channel [3, 4, 61–64] where a soft emission of two vector bosons from the incoming protons is followed by the hard $VV \rightarrow hh$ scattering, with $V=W,Z$. In the SM, the VBF inclusive cross section at 14 TeV is around 2 fb – more than one order of magnitude smaller than in gluon fusion. QCD corrections give a 10% increase and have been computed at NLO in Refs. [53, 65] and at NNLO in Ref. [63]. Production in association with *W* or *Z* bosons, known as the Higgsstrahlung process [65–67], or with top quark pairs [68], exhibit even smaller cross sections.

Despite its small rate, Higgs pair production via VBF is quite interesting since even small modifications of the SM couplings can induce a striking increase of the cross section as a function of the di-Higgs mass. Specific models leading to this behaviour are, for instance, those where the Higgs is a composite pseudo-NGB (pNGB) of new strong dynamics at the TeV scale [69]. In these theories, the Higgs anomalous couplings imply a growth of the $VV\rightarrow hh$ cross section with the partonic centre-of-mass energy, $\hat{\sigma } \propto \hat{s}/f^4$, where *f* is the pNGB decay constant [3]. This enhanced sensitivity to the underlying strength of the Higgs interactions makes double Higgs production via VBF a key process to test the nature of the electroweak symmetry breaking dynamics and to constrain the *hhVV* quartic coupling. A first study of double Higgs production via VBF at the LHC was performed in Ref. [4], for a mass $m_{h}=180\,$GeV, by focusing on the 4*W* final state. Following the discovery of the Higgs boson, more studies of the *hhjj* process at the LHC were presented in Refs. [61, 62, 64, 70].

In this work, we revisit the feasibility of VBF Higgs pair production at the LHC and focus on the $hh\rightarrow b\bar{b}b\bar{b}$ final state. While this final state benefits from increased signal yields due to the large branching fraction of Higgs bosons to bottom quarks, $\mathrm{BR}(H\rightarrow b\bar{b})=0.582$ in the SM [35], it also suffers from overwhelming large QCD multijet backgrounds. In this respect, the remarkable VBF topology, characterized by two forward jets well separated in rapidity and with a large invariant mass, together with a reduced hadronic activity in the central region, provides an essential handle to disentangle signal events from the QCD background. Additionally, the di-Higgs system will acquire a substantial boost in the presence of BSM dynamics. It is thus advantageous to resort to jet-substructure techniques [71] in order to fully exploit the high-energy limit and optimize the signal significance.

We will thus focus on the kinematic region where the invariant mass of the Higgs pair, $m_{hh}$, is large because modifications of the couplings between the Higgs and vector bosons cause the tail of this distribution to become harder in the signal whereas the background is not modified. Therefore, this region exhibits the highest sensitivity to the modified Higgs couplings and in particular to the deviations in the *hhVV* quartic coupling $c_{2V}$. Given that for large $m_{hh}$ the Higgs bosons can be produced boosted, improved discrimination can be achieved using jet substructure, and to this end we use scale-invariant tagging [10, 43] to smoothly combine the resolved, intermediate and boosted topologies.

Our analysis takes into account all the main reducible and irreducible backgrounds: QCD multijet production, Higgs production via gluon fusion (where additional radiation can mimic the VBF topology), and top-quark pair production. We pay special attention to the role of light and charm jets being misidentified as *b*-jets which can contribute sizeably to the total background yield. For instance, in the $gg\rightarrow hh\rightarrow b\bar{b}b\bar{b}$ channel, the 2*b*2*j* background is comparable to the 4*b* component [43].

We quantify the constraints on the Higgs quartic coupling $c_{2V}$ that can be obtained from VBF di-Higgs production at the LHC 14 TeV with $\mathscr {L}=300$ and $3000\,\text {fb}^{-1}$ as well as at a future circular collider (FCC) with a centre-of-mass energy of 100 TeV and a total luminosity of $10\,\text {ab}^{-1}$. We find that, despite the smallness of the production cross sections, the LHC with $300\,\text {fb}^{-1}$ can already constrain the *hhVV* coupling with an accuracy of $_{-37\%}^{+45\%}$ around its SM value at the 1-$\sigma$ level, which is further reduced to $_{-15\%}^{+19\%}$ at the HL-LHC and down to the 1% level at the FCC. Our results strongly motivate that searches for VBF Higgs pair production at the LHC should already start during Run II.

The structure of this paper is as follows. In Sect. 2 we present the general parametrization of the Higgs couplings which we adopt and review its impact on VBF Higgs pair production. Then in Sect. 3, we discuss the analysis strategy used to disentangle the signal from the background events in the $b\bar{b}b\bar{b}$ final state. Our main results are presented in Sect. 4, where we quantify the potential of the VBF di-Higgs process to measure the *hhVV* coupling at various colliders and discuss the validity of the effective field theory expansion. Finally in Sect. 5, we conclude and discuss how our analysis strategy could be applied to related processes. Technical details are collected in three appendices which describe the Monte Carlo event generation of signal and background events (Appendix A), the fits to the tail of the $m_{hh}$ distribution for backgrounds (Appendix B), and the validation studies of the QCD multijet event generation (Appendix C).

**Fig. 1:**
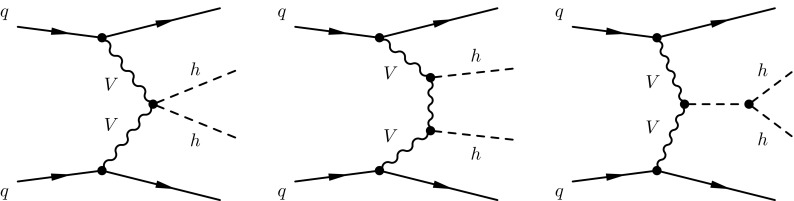
Tree-level Feynman diagrams contributing to Higgs pair production via VBF. In terms of Eq. (2), the *left*, *middle*, and *right diagrams* scale with $c_{2V}$, $c_V^2$, and $c_{V}c_3$, respectively

## Higgs pair production via vector-boson fusion at hadron colliders

We begin by reviewing the theoretical framework for Higgs pair production via vector-boson fusion in hadronic collisions. First, we introduce a general parametrization of the Higgs couplings in the effective field theory (EFT) framework. Then we consider the values that these couplings take in specific models. Finally, we briefly discuss the validity of the EFT approximation and the possible contribution of heavy resonances to this process.

### General parametrization of Higgs couplings

Following Ref. [4], we introduce a general parametrization of the couplings of a light Higgs-like scalar *h* to the SM vector bosons and fermions. At energies much lower than the mass scale of any new resonance, the theory is described by an effective Lagrangian obtained by making a derivative expansion. Under the request of custodial symmetry the three NGBs associated with electroweak symmetry breaking parametrize the coset *SO*(4) / *SO*(3). They can be fitted into a $2\times 2$ matrix

1 $$\begin{aligned} \Sigma =e^{i\sigma ^a\pi ^a/v} \, , \end{aligned}$$

with $v=246\,$GeV the Higgs vacuum expectation value. Assuming that the couplings of the Higgs boson to SM fermions scale with their masses and do not violate flavour, the resulting effective Lagrangian in [4] can be parametrized as

2 $$\begin{aligned} {\mathscr {L}}&\supset \frac{1}{2}(\partial _\mu h)^2 - V(h) +\frac{v^2}{4}\mathrm{Tr}\big (D_\mu \Sigma ^\dagger D^\mu \Sigma \big )\nonumber \\&\quad \times \, \left[ 1+2c_V\, \frac{h}{v}+c_{2V}\,\frac{h^2}{v^2}+\cdots \right] \nonumber \\&\quad -\, m_i\,\bar{\psi }_{Li}\, \Sigma \left( 1+c_{\psi }\,\frac{h}{v} + \cdots \right) \psi _{Ri}\,+\,\mathrm{h.c.}, \end{aligned}$$

where *V*(*h*) denotes the Higgs potential,

3 $$\begin{aligned} V(h) = \frac{1}{2} m_h^2 h^2 + c_3\, \frac{1}{6} \left( \frac{3m_h^2}{v} \right) h^3 + c_4\, \frac{1}{24} \left( \frac{3m_h^2}{v^2} \right) h^4 + \cdots \end{aligned}$$

The parameters $c_V$, $c_{2V}$, $c_{\psi }$, $c_3$, and $c_4$ are in general arbitrary coefficients, normalized so that they equal 1 in the SM. The Higgs mass is fixed to be $m_h=125$ GeV [72].

As the notation in Eq. (2) indicates, the coefficients $c_V$, $c_{2V}$, and $c_3$ control the strength of the *hVV*, *hhVV* and *hhh* couplings, respectively. The coefficients $c_{\psi }$ and $c_4$ instead modify the Higgs coupling to fermions and quartic self interaction. Thus, they do not affect the double-Higgs production cross section in the VBF channel. In Fig. 1, we show the tree-level Feynman diagrams, in the unitary gauge, that contribute to Higgs pair production in the vector-boson fusion channel at hadron colliders. In terms of the general parametrization of Eq. (2), the left, middle, and right diagrams scale with $c_{2V}$, $c_V^2$, and $c_{V}c_3$, respectively.

**Table 1:** Coefficients of Eqs. (7) and (9) as obtained through a fit of Montecarlo points. The cuts are listed in Table 2 and Eqs. (15)–(17)

$\sqrt{s}$	Cuts	$\sigma _\text {sm}$ (fb)	*A*	*B*	*C*	*D*
$14\,$TeV	Acceptance	0.010	$-5.19$	29.5	$-0.939$	0.854
	All	0.0018	$-8.18$	67.5	$-0.699$	0.325
$100\,$TeV	Acceptance	0.20	$-9.18$	306	$-0.699$	0.584
	All	0.030	$-20.7$	1080	$-0.516$	0.251

In the SM, a cancellation dictated by perturbative unitarity occurs between the first and second diagrams. This is best understood by describing the process as a slow emission of the vector bosons by the protons followed by their hard scattering into a pair of Higgs bosons [73]. For generic values of $c_V$ and $c_{2V}$, the amplitude of the partonic scattering $VV\rightarrow hh$ grows with the energy $\sqrt{\hat{s}}$ until the contribution from the new states at the cutoff scale $\Lambda$ unitarizes it. The leading contribution in the energy range $m_W \ll \sqrt{\hat{s}} \equiv m_{hh} \ll \Lambda$ comes from the scattering of longitudinal vector bosons and is given by

4 $$\begin{aligned} {\mathscr {A}}({V_L V_L\rightarrow hh}) \simeq \frac{\hat{s}}{v^2}(c_{2V}-c_V^2), \end{aligned}$$

up to $\mathscr {O}(m_W^2/\hat{s})$ and $\mathscr {O}(\hat{s}/\Lambda ^2)$ corrections. In scenarios with $c_{2V} \ne c_V^2$, the growth of the partonic cross section with $\hat{s}$ thus provides a smoking-gun signature for the presence of BSM dynamics [3].

In the parametrization of Eq. (2), the amplitude for the process $pp \rightarrow hh jj$ can be decomposed as follows:

5 $$\begin{aligned} \mathscr {A} \, = \, \widetilde{A}\,c_V^2 + \widetilde{B}\,c_{2V} + \widetilde{C}\,c_Vc_3 \,, \end{aligned}$$

where $\widetilde{A}$, $\widetilde{B}$, and $\widetilde{C}$ are numerical coefficients. In the present work, we will focus on the quartic coupling $c_{2V}$ and set $c_V$ and $c_3$ to their SM values. This is justified for $c_V$ since the ATLAS and CMS measurements of Higgs production cross sections, when analyzed in the context of a global fit of Higgs properties [74–76] typically set bounds on $c_V-1$ at the level of 10–20%, depending on the specific assumptions made – see for example [77–79] and the references therein. Tighter limits on $c_V$ can be derived from electroweak precision tests in the absence of additional BSM contributions [80].

On the other hand, the trilinear Higgs coupling $c_3$ (where $c_3 = \lambda /\lambda _{\text {sm}}$) only has loose experimental constraints so far. As an illustration, a recent ATLAS search for non-resonant Higgs pair production at 13 TeV in the $b\bar{b}b\bar{b}$ final state [27] translates into the bound $\sigma (hh)/\sigma _\text {sm}(hh)\lesssim 27$ at the 95% confidence level. Achieving $\mathscr {O}(1)$ precision in the measurement of $c_3$ will thus most likely require the full HL-LHC statistics. Focusing on VBF production, as anticipated and further discussed in the following, gaining sensitivity to $c_{2V}$ is achieved by reconstructing events with large values of $m_{hh}$. In this kinematic region, it turns out that the sensitivity to $c_3$ is reduced, indicating that our analysis is not optimal to probe the Higgs trilinear coupling. For these reasons, setting $c_V = c_3 =1$ is a good approximation in the context of the present analysis. We can then define

6 $$\begin{aligned} \delta _{c_{2V}} \equiv c_{2V}-1, \end{aligned}$$

and this way the total cross section will be parametrized as

7 $$\begin{aligned} \sigma = \sigma _\text {sm}\big (1+A\,\delta _{c_{2V}}+ B\,\delta _{c_{2V}}^2\big ). \end{aligned}$$

However, while setting $c_3=1$ is a very good approximation, fixing $c_V=1$ is not as equally well justified. In particular, it would be more prudent to treat $c_V$ as a Gaussian distributed nuisance parameter centered around its SM value with a width corresponding to the current experimental precision. To do this, a similar expression to Eq. (7) above can be derived by neglecting the subleading effects involving $c_3$. In this case, Eq. (7) is replaced by

8 $$\begin{aligned} \sigma \approx \sigma _\text {sm}\,c_V^4\left( 1+A\,\left[ \frac{c_{2V}}{c_V^2}-1\right] + B\,\left[ \frac{c_{2V}}{c_V^2}-1\right] ^2\right) . \end{aligned}$$

We will use this expression to evaluate the impact of $c_V$ on the derived bounds on $\delta _{c_{2V}}$ at the end of Sect. 4. The values of the SM cross section $\sigma _\text {sm}$ and of the parameters *A*, *B* are reported in Table 1 for $\sqrt{s}=14$ and 100 TeV, both after acceptance cuts and after applying all the analysis cuts as discussed in Sect. 3 – see Appendix D for the values of the parameters in bins of $m_{hh}$. We will make extensive use of this parametrization in Sect. 4 where we present our results in terms of the sensitivity on $\delta _{c_{2V}}$. Note that the value of *A* and *B* increase after imposing all cuts precisely because they have been optimized to enhance the sensitivity on $c_{2V}$.

Although we do not attempt to extract $c_3$ with our analysis, it is still interesting to discuss the dependence of the total cross section on this parameter. By fixing $c_V = c_{2V} =1$ and defining $\delta _{c_3}\equiv c_3-1$, the cross section can now be parametrized as

9 $$\begin{aligned} \sigma = \sigma _\text {sm}\big (1+C\,\delta _{c_3} + D\,\delta _{c_3}^2\big ). \end{aligned}$$

The coefficients *C* and *D* are also reported in Table 1. As opposed to the previous case, now their values decrease after applying the full set of cuts, reflecting that the sensitivity on $c_3$ is suppressed by our analysis which aimed at measuring $c_{2V}$. Extracting $c_3$ would require retaining the events close to the *hh* threshold but this kinematic region is totally dominated by the background and turns out to be of little use. A measurement of the Higgs trilinear coupling in the VBF channel using the $b\bar{b} b \bar{b}$ final state thus does not seem feasible even at the FCC. However, other final states might exhibit better prospects at 100 TeV.

### Models

The Lagrangian of Eq. (2), with arbitrary values of the coefficients $c_V$, $c_{2V}$, $c_{\psi }$, $c_3$ and $c_4$, describes a generic light scalar, singlet of the custodial symmetry, independently of its role in the electroweak symmetry breaking. In specific UV models, however, the coefficients $c_i$ are generally related to each other and their values are subject to constraints which depend on whether the Higgs-like boson *h* is part of an $SU(2)_L$ doublet. For example, in the SM, all the parameters in Eq. (2) are equal to 1 and terms denoted by the ellipses vanish. In this case the scalar *h* and the three NGBs combine to form a doublet of $SU(2)_L$ which is realized linearly at high energies.

Composite Higgs theories are another example where the electroweak symmetry is realized linearly in the UV, though in this case non-linearities in the Higgs interactions can be large and are controlled by the ratio $\xi \equiv v^2/f^2$, where *f* is the pNGB decay constant. For instance, minimal *SO*(5) / *SO*(4) models [81, 82] predict

10 $$\begin{aligned} c_V=\sqrt{1-\xi },\qquad c_{2V}=1-2\xi . \end{aligned}$$

On the other hand, the value of the Higgs trilinear coupling is not determined by the coset structure alone, and depends on how the Higgs potential is specifically generated. For instance, in the MCHM5 model with fermions transforming as vector representations of *SO*(5) [82], the Higgs potential is entirely generated by loops of SM fields and the Higgs trilinear coupling is predicted to be

11 $$\begin{aligned} c_3 = \frac{1-2\xi }{\sqrt{1-\xi }}. \end{aligned}$$

A precision model-independent determination of $c_{2V}$ would thus provide stringent constraints on a number of BSM scenarios. To begin with, if the Higgs boson belongs to an electroweak doublet, as suggested by the LHC data, and the modifications to its couplings are small, then the values of $c_{2V}$ and $c_{2V}$ are in general predicted to be correlated [3, 5]:

12 $$\begin{aligned} \delta _{c_{2V}} \simeq 2\, \delta _{c_V^2}, \end{aligned}$$

where $\delta _{c_V^2} \equiv c_V^2 -1$. This follows because there is a single dimension-6 effective operator ($O_H$ in the basis of Ref. [3]) which controls the shift in both couplings. Therefore, a high-precision measurement of $c_{2V}$ can test whether the Higgs boson belongs to a doublet in case a deviation is observed in $c_V$ [5].

Another interesting case is the scenario where the Higgs-like boson is not part of a doublet, and in fact does not play any role in the electroweak symmetry breaking mechanism, known as the light dilaton scenario [83–88]. In this model, invariance under dilatations implies $\delta _{c_{2V}} = \delta _{c_V^2}$, a condition that can be tested if the two couplings in Eq. (12) can be measured with comparable precision. Moreover, comparing $c_{2V}$ and $c_V$ can also provide information on the coset structure in the case of a composite NGB Higgs [5].

**Fig. 2:**
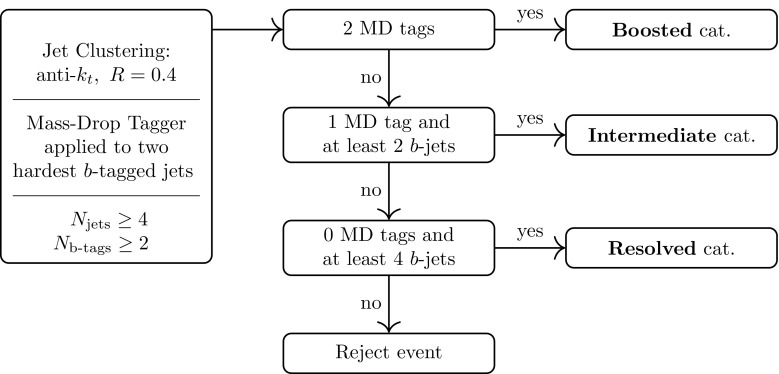
Schematic representation of the analysis strategy adopted in this work

A large variety of BSM scenarios also exists where the Higgs trilinear coupling receives large modifications while the value of the other couplings are close to the SM prediction. Higgs portal models fall in this class; see for example the discussion in [17, 36] and also [89, 90]. However, since our analysis is not sensitive to $c_3$, we will not consider these scenarios any further and will always assume $\delta _{c_3}=0$.

In the following, we will take as a representative benchmark scenario a model with $c_{2V}=0.8$ corresponding to $\delta _{c_{2V}}=-0.2$, with the other couplings set to their SM values, namely $\delta _{c_V}=\delta _{c_3}=0$.

### Validity range of the effective theory

As discussed above and shown by Eq. (4), the amplitude of the partonic scattering $VV\rightarrow hh$ grows with the energy in the EFT described by Eq. (2). However, this behaviour holds only below the typical mass scale of new BSM states, i.e. below the cutoff scale $\Lambda$ of the effective theory. When the invariant mass of the di-Higgs system becomes large enough, the EFT approximation breaks down and it becomes necessary to take into full account the contribution from the exchange of new states, such as vector and scalar resonances. These resonances eventually tame the growth of the scattering amplitude at large energies to be consistent with perturbative unitarity bounds. In the context of composite Higgs scenarios the impact of resonances on *VV* scattering has been explored for instance in [91–96].

While we do not include the effect of such resonances in this work, we will report the sensitivity on $\delta _{c_{2V}}$ as a function of the maximum value of the invariant mass of the di-Higgs system (see Fig. 13 in Sect. 4), as suggested by Ref. [97]. This comparison allows one to assess the validity of the EFT description once an estimate of $c_{2V}$ is provided in terms of the masses and couplings of the UV dynamics. The result of this analysis – which is discussed in detail at the end of Sect. 4 – confirms that the EFT is valid over the full range of $m_{hh}$ that is used to derive limits on $\delta _{c_{2V}}$. The explicit inclusion of scalar and vector resonances and their phenomenological implications for Higgs pair production via VBF is left for future work.

## Analysis strategy

In this section we present our analysis of double Higgs production via VBF. First, we discuss how signal and background events are reconstructed and classified. This includes a description of the jet reconstruction techniques adopted, the *b*-tagging strategy, and the event categorization in terms of jet substructure. Then we illustrate the various selection cuts imposed to maximize the signal significance, in particular the VBF cuts, as well as the method used to identify the Higgs boson candidates. Finally, we present the signal and background event rates for the various steps of the analysis, and discuss how the signal cross sections are modified when $c_{2V}$ is varied as compared to its SM value.

### Event reconstruction and classification

Signal and background events are simulated at leading order (LO) by means of matrix-element generators and then processed through a parton shower (PS). The detailed description of the event generation of signal and backgrounds can be found in Appendix A. The dominant background is given by QCD multijet production, while other backgrounds, such as top-quark pair production and Higgs pair production via gluon fusion, are much smaller. After the parton shower, events are clustered with FastJet v3.0.1 [98] using the anti-$k_t$ algorithm [99] with a jet radius $R=0.4$.

The resulting jets are processed through a *b*-tagging algorithm, where a jet is tagged as *b*-jet with probability $\varepsilon (b\text {-tag})$ if it contains a *b*-quark with $p_T^b > 15\,$GeV. In order to account for *b*-jet misidentification (fakes), jets which do not meet this requirement are also tagged as *b*-jets with probability $\varepsilon (c\text {-mistag})$ or $\varepsilon (q,g\text {-mistag})$ depending on whether they contain a *c*-quark or not. Only events with four or more jets, of which at least two must be *b*-tagged, are retained at this stage.

Fully exploiting the $b\bar{b}b\bar{b}$ final state requires efficient *b*-tagging capabilities in both the resolved and boosted regimes as well as a good rejection of fakes. Both ATLAS and CMS have presented recent studies of their capabilities in terms of *b*-tagging and light-jet fake rejection for both topologies; see Refs. [100–104] and the references therein. In the present study, we have considered two representative *b*-tagging working points:

13 $$\begin{aligned}&\text {WP1}:\; \varepsilon (b\text {-tag})=0.75, \quad \varepsilon (c\text {-mistag}) = 0.1, \nonumber \\&\qquad \varepsilon (q,g\text {-mistag}) = 0.01, \nonumber \\&\text {WP2}:\; \varepsilon (b\text {-tag})=0.8, \quad \varepsilon (c\text {-mistag}) = 0.05, \nonumber \\&\qquad \varepsilon (q,g\text {-mistag}) = 0.005. \end{aligned}$$

The first point is consistent with the current performance of ATLAS and CMS, and is the one adopted as baseline in this paper. The second working point is more optimistic and is intended to assess how much one could gain with a more efficient *b*-tagger. As our results will show (see Fig. 14), using WP2 leads to a marginal improvement in our analysis. For simplicity, we applied the efficiencies in Eq. (13) to a jet based on its constituents. This is sufficient for the purpose of the current analysis, which is namely to demonstrate the sensitivity of double Higgs production via VBF to $\delta _{c_{2V}}$. Accordingly, we leave a detailed study of *b*-tagging including hadronization effects and $p_T$ dependence to future studies.

Subsequently to *b*-tagging, events are classified through a scale-invariant tagging procedure [10, 43]. This step is crucial to efficiently reconstruct the Higgs boson candidates and suppress the otherwise overwhelming QCD backgrounds while at the same time taking into account all the relevant final-state topologies. The basic idea of this method is to robustly merge three event topologies – *boosted, intermediate* and *resolved* – into a common analysis. This is particularly relevant for our study, since, as discussed in Sect. 2, the degree of boost of the di-Higgs system strongly depends on the deviations of $c_{2V}$ from its SM value.

This scale-invariant tagging strategy is schematically represented in Fig. 2. First of all, the *b*-tagged jets are ordered in $p_T$ and the constituents of the hardest two jets are then re-clustered using the Cambridge/Aachen (C/A) algorithm [105] with $R_\mathrm{C/A}=1.2$. Each C/A jet is processed with the BDRS mass-drop (MD) tagger [106]. This jet-substructure tagger has two parameters: $\mu$ and $y_\mathrm{cut}$. And, in this work, we set $\mu = 0.67$ and $y_\mathrm{cut} = 0.09$ as in the original BDRS study. To determine if a given jet arises from the decay of a massive object, the last step of the clustering for jet *j* is undone, giving two subjets $j_1$ and $j_2$ which are ordered such that $m_{j_1} > m_{j_2}$. Then, if the two subjets satisfy the conditions

14 $$\begin{aligned} m_{j_1}\le \mu \cdot m_j\quad \text {and}\quad \min (p_{Tj_1}^2,p_{Tj_2}^2)\,\Delta R^2_{j_1,j_2} > y_\mathrm{cut}\cdot m_{j}^2, \end{aligned}$$

where $\Delta R_{j_1,j_2}$ is the angular separation between the two subjets, *j* is tagged as a jet with a mass drop. Else, the procedure is applied recursively to $j_1$ until a mass drop is found or the C/A jet is fully unclustered.

Jets are mass-drop tagged only if they satisfy the following additional requirement: at least two *b*-quarks must be contained within the jet, each of which with $p_{Tb}\ge 15\,$GeV, and with a minimal angular separation $\Delta R_{bb}\ge 0.1$. The request of a second *b*-quark completes our *b*-tagging algorithm in the case of boosted jets. Other more sophisticated approaches to *b*-tagging could have been considered – e.g., using ghost-association between large-*R* MD-tagged jets and small-*R* *b*-tagged jets [23, 43], or accounting for an efficiency which depends on the jet $p_T$ [107]. The approach followed here is at the same time simple yet realistic enough for a first feasibility study with the caveat that a more complete analysis should treat *b*-tagging more in line with the actual performance of the ATLAS and CMS detectors (and in particular it should include a full detector simulation).

The use of the BDRS mass-drop tagger allows us to classify a given signal or background event under one of the three categories: boosted, if two mass-drop tags are present; intermediate, for an event with a single mass-drop tag; and resolved, if the event has no mass-drop tags. In the resolved category, events are only retained if they contain at least four *b*-tagged jets, while at least two *b*-tagged jets, in addition to the MD-tagged jet, are required in the intermediate one. By construction, this classification is exclusive, i.e., each event is unambiguously assigned to one of the three categories. This exclusivity allows the consistent combination of the signal significance from the three separate categories.

Following the event categorization, acceptance cuts to match detector coverage are applied to signal and background events. These cuts are listed in the upper part of Table 2, and have been separately optimized for the LHC $14\,$TeV and the FCC 100 TeV. We require the $p_T$ of the light (*b*-tagged) jets to be larger than 25 GeV (25 GeV) at 14 TeV and than 40 GeV (35 GeV) at 100 TeV, respectively. Concerning the pseudo-rapidities of light and *b*-tagged jets, $\eta _j$ and $\eta _b$, at the LHC the former is limited by the coverage of the forward calorimeters, while the latter is constrained by the tracking region where *b*-tagging can be applied. At 100 TeV, we assume a detector with extended coverage of the forward region up to $|\eta |$ of 6.5 [108].

**Table 2:** Acceptance and VBF selection cuts applied to signal and background events after jet clustering and *b*-tagging. The central jet veto is applied on jets with pseudo-rapidity $\eta _{j_3}$ in the interval $\eta _j^{\min }< \eta _{j_3} < \eta _j^{\max }$, where $\eta _j^{\max }$ and $\eta _j^{\min }$ are the pseudo-rapidities of the VBF tagging jets

	14 TeV	100 TeV
Acceptance cuts		
$p_{T_j}~\text {(GeV)}~\ge ~$	25	40
$p_{T_b}~\text {(GeV)}~\ge ~$	25	35
$|\eta _j|\le ~$	4.5	6.5
$|\eta _b|\le ~$	2.5	3.0
VBF cuts		
$|\Delta y_{jj}|\ge ~$	5.0	5.0
$m_{jj}~\text {(GeV)}~\ge ~$	700	1000
Central jet veto: $p_{T_{j_3}}~\text {(GeV)}~\le ~$	45	65

### VBF selection cuts

Subsequently to the acceptance cuts, we impose a set of selection cuts tailored to the VBF topology which is characterized by two forward and very energetic jets with little hadronic activity between them. In particular, we cut on the rapidity separation $\Delta y_{jj}\equiv |y_j^\mathrm{lead}-y_j^\mathrm{sublead}|$ and the invariant mass $m_{jj}$ of the two VBF tagging jets, and impose a central jet veto (CJV) on the hardest non-VBF light jet in the central region. The VBF tagging jets are defined as the pair of light jets satisfying the acceptance cuts of Table 2 with the largest invariant mass $m_{jj}$. This definition is robust with respect to soft contamination from the underlying event (UE) and pile-up (PU) and to the contribution of *b*-jets mistagged as light jets.

**Fig. 3:**
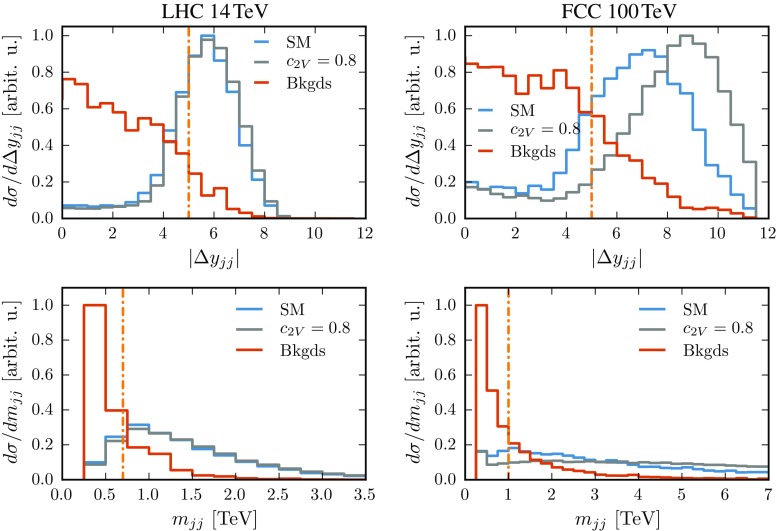
Distribution of the rapidity separation $|\Delta y_{jj}|$ (*upper*) and the invariant mass $m_{jj}$ (*bottom panels*) of the VBF tagging jets at 14 TeV (*left*) and 100 TeV (*right panels*), for signal (SM and $c_{2V}=0.8$) and background events after the acceptance cuts The *vertical line* indicates the value of the corresponding cut from Table 2. The distributions have been area-normalized and rescaled by a common factor

Figure 3 shows the distribution of the rapidity separation $|\Delta y_{jj}|$ and invariant mass $m_{jj}$ of the VBF tagging jets at 14 and 100 TeV after the acceptance cuts. In each case, we show the results for the signal (SM and $c_{2V}=0.8$ benchmark) and for the total background. The signal distributions exhibit the distinctive VBF topology, with two VBF tagging jets widely separated in rapidity and with a large invariant mass. This is in contrast with the backgrounds where both the $\Delta y_{jj}$ and $m_{jj}$ distributions peak at zero. In Fig. 3, as well as in the subsequent figures, kinematic distributions have been area-normalized and then rescaled by a common factor such that the largest bin in the plot is of unit height.

Based on the distributions of Fig. 3 we identified appropriate values of the VBF cuts, listed in Table 2 and represented in each panel by a vertical dash-dotted line. It is important to tailor these cuts to the specific centre-of-mass energy, 14 and 100 TeV, to avoid losing a substantial fraction of the signal events. One should also take into account that the large rapidity separation between the VBF tagging jets in signal events results from jets pairs with a large invariant mass, given that these two variables are strongly correlated [4]. This large separation in rapidity is especially useful in the $b\bar{b}b\bar{b}$ final state to trigger on signal events, providing a significant improvement compared to the same final state produced in gluon fusion where triggering issues are more severe [42, 43].

**Fig. 4:**
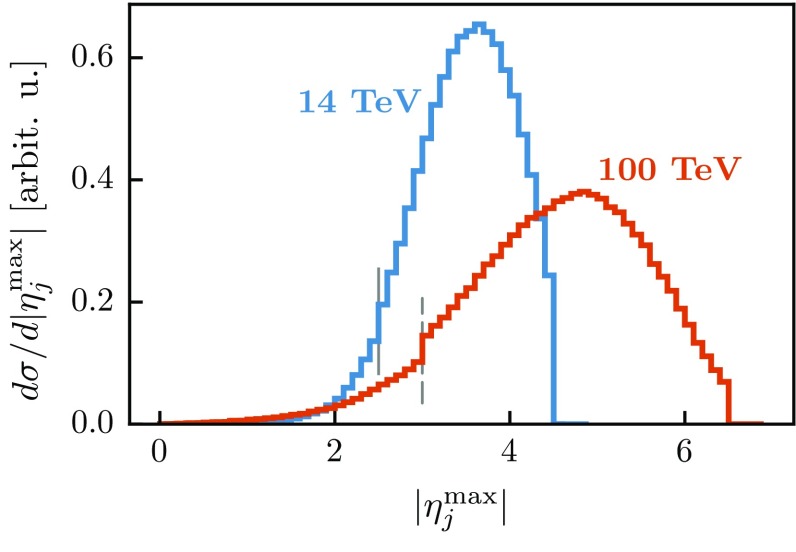
Distribution of the pseudo-rapidity $|\eta _j^\mathrm{max}|$ of the most forward light jet at 14 and 100 TeV. Both curves have a discontinuity at the edge of the corresponding b-tagging region which is delineated by the *solid* (*dashed*) *grey* *vertical lines* in the case of 14 (100) TeV. This discontinuity is more clearly visible in the 100 TeV curve and is purely due to combinatorics

Figure 3 clearly highlights that in order to maximize the acceptance of events with VBF topology – the detectors must have a good coverage of the forward region. This issue is particularly relevant at 100 TeV as illustrated in Fig. 4 which shows the pseudo-rapidity distribution of the most forward light jet. At 100 TeV, this peaks at around 5, so a detector instrumented only up to $|\eta |=4.5$ would lose more than 50% of signal events. The discontinuity in the FCC case delineates the edge of the *b*-jet acceptance region, $|\eta |\le 3$, above which no *b*-tagging is attempted and *b*-jets contribute to the light-jet yield.

**Fig. 5:**
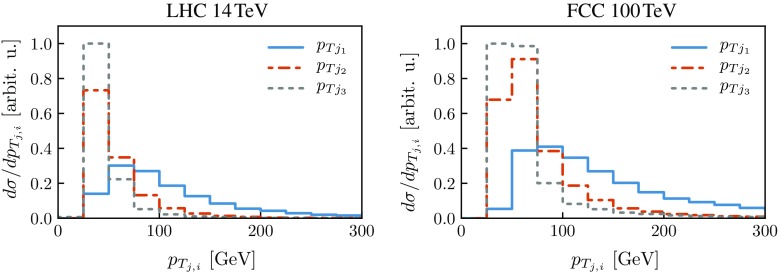
Distributions of the $p_T$ of the leading, subleading, and third light jet at 14 TeV (*left panel*) and 100 TeV (*right panel*) for the SM signal. Distributions are area-normalized as in Fig. 3

Turning to the transverse momentum of the light jets, Fig. 5 shows the $p_T$ distributions of the three hardest jets at 14 and 100 TeV for SM signal events. One can see that while the leading jet is typically quite hard, the subleading ones are rather soft. It is thus important to avoid imposing a too stringent cut in $p_{T_{}}$, in order not to suppress the signal. Fortunately, in contrast from the gluon-fusion process, adopting a soft $p_{T_{}}$ cut is not a problem since triggering can be performed based on the VBF topology. Comparing the $p_{T_{}}$ distributions at 14 and 100 TeV, their shapes turn out to be rather similar, shifted towards larger values at $100\,$TeV. This justifies the harder $p_{T_{}}$ cut in this case (see Table 2), also required to reduce the contamination from UE and PU.

**Fig. 6:**
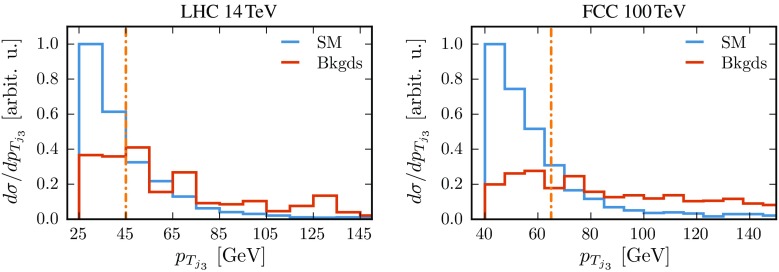
Distribution of the $p_T$ of the third light jet at 14 TeV (*left panel*) and 100 TeV (*right panel*) for the SM signal and the total background, including only events where this jet lies within the pseudo-rapidity region between the VBF jets. The *vertical line* indicates the CJV cut

As mentioned above, another characteristic feature of VBF production is a reduced hadronic activity in the central region between the two VBF tagging jets. This follows because the latter are not colour-connected since the production of the central system only involves electroweak bosons. For this reason, a CJV cut is commonly imposed in VBF analyzes. This cut vetoes light jets, with pseudo-rapidity $\eta _{j_3}$, lying between those of the VBF tagging jets, $\eta _j^{\max }>\eta _{j_3}>\eta _j^{\min }$, above a given $p_{T_{}}$ threshold.

The effect of the CJV is illustrated in Fig. 6 where we show the distribution of the $p_T$ of the third light jet, $p_{T_{j_3}}$, for the SM signal and the total background. Although the latter has a harder spectrum than the signal, imposing too stringent a veto is not advantageous. This is because the $b\bar{b}b\bar{b}$ final state leads to a non-negligible amount of hadronic activity in the central region, for instance due to gluon radiation from the *b* quarks and to *b*-jet misidentification. Based on these results, in our analysis, we impose a CJV with the threshold value reported in Table 2 and shown in the plots by the dot-dashed line.

### Higgs reconstruction

The next step in our analysis is the reconstruction of the Higgs boson candidates. This is done separately for each of the three event categories. In the resolved category, starting with the six hardest *b*-jets in the event,^1^ we reconstruct the first Higgs boson candidate $h_1$ by identifying it with the pair of *b*-jets whose invariant mass is closest to the Higgs mass, $m_h=125\,$GeV. Out of the remaining *b*-jet pairs, the one with an invariant mass closest to $m_{h_1}$ is then assigned to be the second Higgs boson candidate, $h_2$. In the case of the intermediate and boosted categories, each of the mass-drop tagged jets is identified with a Higgs candidate. The second Higgs candidate in the intermediate category is then formed by considering the five hardest *b*-jets in the event and selecting the pair whose mass is closest to $m_h$.

**Fig. 7:**
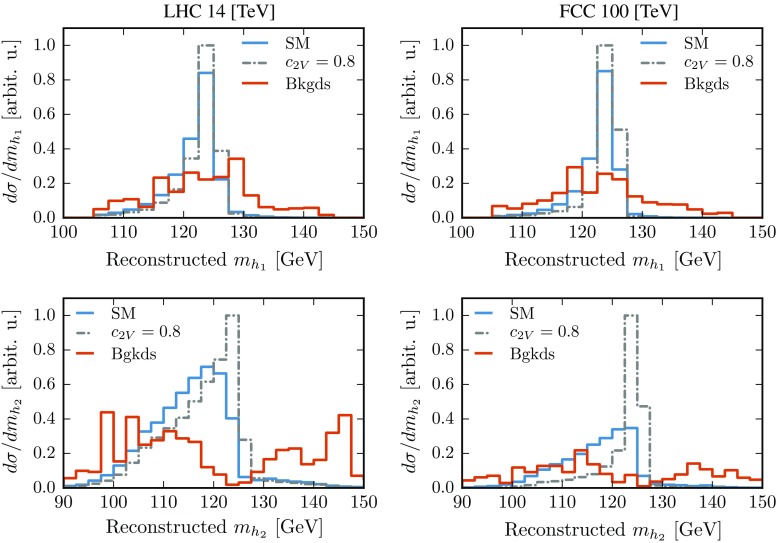
Invariant mass distribution of the leading ($m_{h_1}$) and subleading ($m_{h_2}$) Higgs candidates for signal (SM and $c_{2V}=0.8$) and background events at 14 TeV (*left*) and 100 TeV (*right*)

**Fig. 8:**
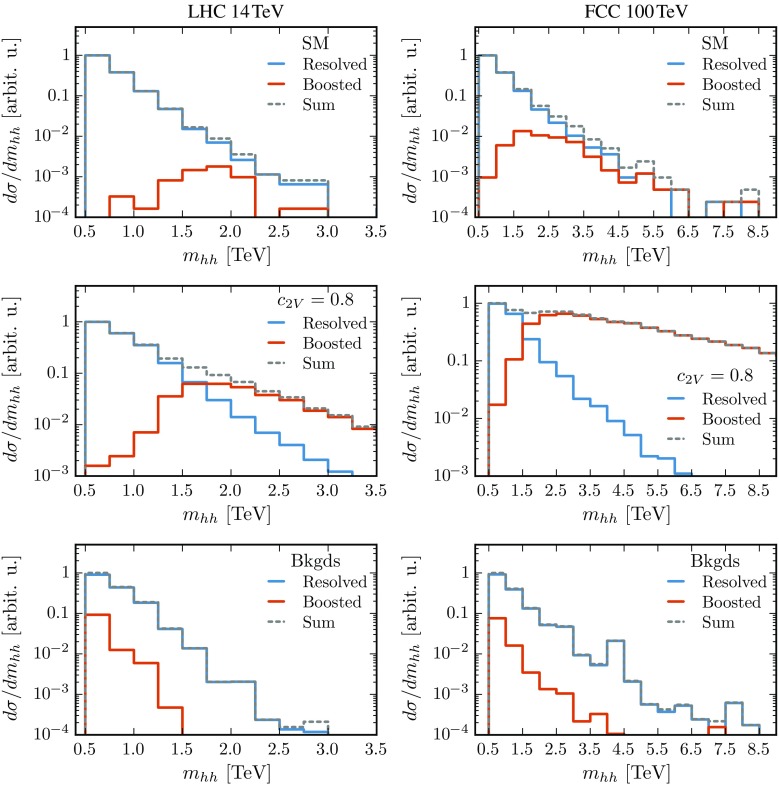
Invariant mass distribution of the di-Higgs system at 14 TeV (*left*) and 100 TeV (*right*) after all analysis cuts, for the signal (SM and $c_{2V}=0.8$) and the total background. We show the contribution from resolved and boosted events as well as the sum of the three categorieslabelfig

The invariant mass distributions of the Higgs candidates for the signal (SM and $c_{2V}=0.8$) and the total background are shown in Fig. 7. The peak around $m_h = 125\,$GeV is clearly visible for signal events, especially in the case of $h_1$. The smearing of the signal distribution of the second Higgs candidate $h_2$ arises from out-of-cone radiation effects which reduce the reconstructed mass. It is largest in the SM, while it is reduced in the $c_{2V}=0.8$ scenario and in particular at $100\,$TeV, due to the larger boost of the Higgs bosons. The small peak in the background distributions for $h_1$ is artificially sculpted by the analysis selection cuts. The fact that the efficiency for the reconstruction of the Higgs bosons is similar in the SM and for the $c_{2V}=0.8$ benchmark is another validation of the scale-invariant tagging, since while in the SM most events lead to resolved topologies, the $c_{2V}=0.8$ scenario is dominated by the boosted category (see Fig. 8 below).

After reconstructing the Higgs candidates, we require that their invariant masses, $m_{h_1}$ and $m_{h_2}$, are reasonably close to the nominal mass. In the resolved category, these conditions are

15 $$\begin{aligned}&|m_{h_1}- 125\,\text {GeV}|~\le ~20~\mathrm{GeV},\end{aligned}$$

16 $$\begin{aligned}&|m_{h_2}-m_{h_1}|~\le ~20~\mathrm{GeV}. \end{aligned}$$

The mass window in Eq. (16) is centered around the mass of the first candidate rather than the nominal Higgs mass in order to make the cut robust against UE and PU effects. For the intermediate and boosted categories, the invariant mass of all Higgs candidates is required to satisfy Eq. (15). The cuts of Eqs. (15) and (16) are especially effective in suppressing the QCD backgrounds which have almost featureless $m_{h_i}$ distributions in the Higgs mass regions. Finally, we impose an additional cut on the invariant mass of the di-Higgs system:

17 $$\begin{aligned}&\text {LHC 14 TeV}:\; m_{hh}>500\,\text {GeV},\nonumber \\&\text {FCC 100 TeV}:\; m_{hh}>1000\,\text {GeV}. \end{aligned}$$

This condition greatly reduces the background rates while leaving the interesting kinematic region at large $m_{hh}$ – where deviations from the SM signal mostly appear – unaffected.

Figure 8 shows the $m_{hh}$ distribution, after all the cuts listed in Table 2 and Eqs. (15)–(17), for signal (SM and $c_{2V}=0.8$) and the total background at 14 and 100 TeV. In each case, we show both the sum of the three event categories and the individual contributions from resolved and boosted events. The intermediate category, which contributes very little in all cases, is not shown. This comparison helps illustrate the relative weight of the boosted and resolved categories. For signal events in the SM, the vast majority are classified in the resolved category as expected since in this case the boost of the di-Higgs system is small except at 100 TeV and for large $m_{hh}$ values. On the other hand, in the case of $c_{2V}=0.8$, the energy growth of the partonic cross section induces a much harder $m_{hh}$ spectrum. This implies that, already at 14 TeV, a substantial fraction of events falls in the boosted category which becomes the dominant one at 100 TeV. For $c_{2V}=0.8$, the crossover between the resolved and boosted categories takes place at $m_{hh}\simeq 1.5$ TeV for both colliders, although this specific value depends on the choice of the jet radius *R* [10]. Unsurprisingly, background events are always dominated by the resolved topology.

### Signal and background event rates

Now that we have presented our analysis strategy we can discuss the actual impact on the cross sections and event rates of the various steps of the cut flow. In Table 3 we report the cross sections at 14 and 100 TeV after acceptance, VBF, Higgs reconstruction, and $m_{hh}$ cuts of Table 2 and Eqs. (15)–(17), respectively, for both the signal (SM and $c_{2V}=0.8$) and for the total background.

At $14\,$TeV, we find that the VBF di-Higgs signal in the SM is rather small already after the basic acceptance cuts. On the other hand, the signal event yield is substantially increased for $c_{2V}\ne 1$ as illustrated by the benchmark value of $c_{2V}= 0.8$ leading to more than a factor 3 (5) enhancement compared to the SM after the acceptance (all analysis) cuts. The fact that this cross-section enhancement for the $c_{2V} = 0.8$ scenario is more marked at the end of the analysis is not a coincidence: our selection cuts have been designed so as to improve the sensitivity to $c_{2V}$ by increasing the signal significance in the large-$m_{hh}$ region.

From Table 3 we also find that a similar qualitative picture holds at $100\,$TeV with the important difference that, in this case, the event rate is already substantial in the SM which yields ${\simeq } 2000$ events after the acceptance cuts with $\mathscr {L}=10\,$ab$^{-1}$. The cross section enhancement at $100\,$TeV as compared to $14\,$TeV is driven by the larger centre-of-mass energy and leads to a signal rate greater by a factor 20 (17) after the acceptance (all analysis) cuts in the SM, and by a factor ${\simeq } 100\,(150)$ in the $c_{2V}=0.8$ scenario. At $100\,$TeV, the ratio of signal cross sections in the $c_{2V}=0.8$ and SM scenarios is ${\sim } 15\,(50)$ after acceptance (all analysis) cuts. Note however, that at both 14 and 100 TeV, even after all analysis cuts the background is still much larger than the signal (either SM or $c_{2V}=0.8$) at the level of inclusive rates. It is only by exploiting the large-$m_{hh}$ region that the former can be made small enough to achieve high signal significances.

**Table 3:** Cross sections, in fb, at $14\,$TeV (upper table) and $100\,$TeV (lower table) after the successive application of the acceptance and VBF cuts (Table 2) and of the Higgs reconstruction cuts (Eqs. (15)–(17)), for signal events (SM and $c_{2V}=0.8$) and for the total background

	Cross sections (fb)			
	Acceptance	VBF	Higgs reco.	$m_{hh}$ cut
14 TeV				
Signal SM	0.011	0.0061	0.0039	0.0020
Signal $c_{2V}=0.8$	0.035	0.020	0.017	0.011
Bkgd (total)	$1.3\times 10^{5}$	$4.9\times 10^{3}$	569	47
100 TeV				
Signal SM	0.22	0.15	0.11	0.033
Signal $c_{2V}=0.8$	3.4	2.7	1.9	1.6
Bkgd (total)	$1.9\times 10^{6}$	$1.9\times 10^{5}$	$9.5\times 10^{3}$	212

**Fig. 9:**
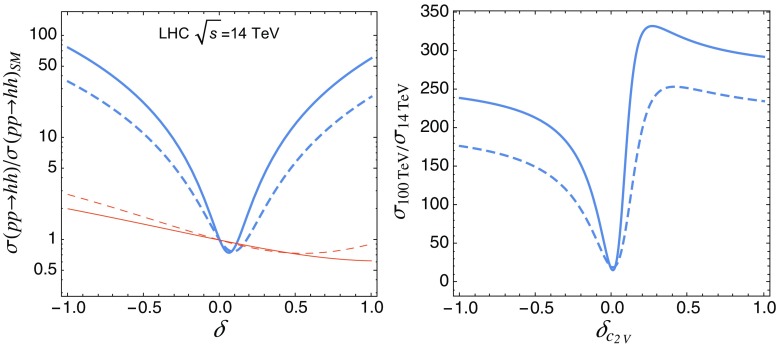
*Left panel* VBF di-Higgs cross section, in units of the SM value, as a function of $\delta _{c_{2V}}$ (*thick blue*) and $\delta c_{3}$ (*thin red*), after acceptance cuts (*solid*) and all analysis cuts (*dashed*). *Right panel* ratio of VBF di-Higgs cross section between 100 and 14 TeV as a function of $\delta _{c_{2V}}$

Table 3 is also useful to assess the relative impact on the signal and the total background of each of the cuts imposed. In the case of the VBF cuts, we find that the background is drastically reduced, by more than one order of magnitude, at the cost of a moderate decrease of the signal cross sections. The Higgs mass window requirement is also instrumental to further suppress the backgrounds, especially the QCD multijets which are featureless in $m_h$, while leaving the signal mostly unaffected. A final reduction of the background, by around another order of magnitude, is achieved through the $m_{hh}$ cut. The relative impact of each cut on signal events is similar in the SM and for $c_{2V}=0.8$.

Figure 9 graphically illustrates the dependence of the di-Higgs production cross section on the couplings $c_{2V}$ and $c_{3}$. The left panel shows the cross section in SM units as a function of $\delta _{c_{2V}}=c_{2V}-1$ and $\delta _{c_{3}}=c_{3}-1$ after applying the acceptance cuts of Table 2 (dashed curves) and after all the analysis cuts (solid curves). The sensitivity on $c_{2V}$ is particularly striking, for example the cross section for $|\delta _{c_{2V}}|\simeq 1$ is enhanced by a factor ${\sim } 50$ compared to its SM value after all cuts. This sensitivity is the key ingredient for measuring $c_{2V}$ with good precision, even though the SM cross section itself cannot be extracted with comparable accuracy. In particular, as we will show in Sect. 4, the sensitivity to $\delta _{c_{2V}}$ derives mainly from the tail of the $m_{hh}$ distribution. This observation elucidates the enhancement (suppression) in the sensitivity to $\delta _{c_{2V}}\,(\delta _{c_3})$ in Fig. 9 after the application of all the cuts which, for instance, remove the threshold region up to $m_{hh}=500\,(1000)$ GeV at the LHC(FCC).

The right panel of Fig. 9 shows, instead, the ratio between the VBF di-Higgs cross sections at $\sqrt{s}=100$ and 14 TeV. Given the larger centre-of-mass energy of the FCC, it is expected that this ratio grows rapidly for $\delta _{c_{2V}}\ne 0$ and, indeed, it can reach values as high as 300 for $\delta _{c_{2V}}\simeq 1$. As will be demonstrated in Sect. 4, this effect allows for much more precise measurements of $c_{2V}$ at the FCC, with uncertainties reduced by a factor 20 as compared to the HL-LHC. The results of Fig. 9 are of course consistent with the findings of Table 3.

From Fig. 9, we also observe that the sensitivity of the signal on the Higgs trilinear coupling $c_3$ is relatively weak even for large variations and it is reduced by our analysis strategy. As already mentioned, the last feature is expected because the sensitivity to $c_{3}$ comes from events near the di-Higgs threshold, $m_{hh}\simeq 2m_{h}$, which are removed by our cuts due to the overwhelming backgrounds in that region. This weak dependence of the VBF di-Higgs cross section on $c_3$, together with the large event rates for background after all the analysis cuts (see Table 3), suggest that the VBF process is not suitable to extract the Higgs self-coupling.

Let us now discuss the background processes. As mentioned above and discussed in Appendix A, there are two types of processes that contribute to the final-state signature under consideration. The first type are QCD processes and in particular multijet and top-quark pair production in association with additional hard radiation. The second is Higgs pair production in the gluon–gluon fusion channel in association with additional jets, where the latter can mimic the VBF topology, as in single-Higgs production.

In the case of QCD multijet processes, it is important to account for the effects of both the 4*b* and the 2*b*2*j* backgrounds (where we label each process by its matrix-element level content; as explained in Appendix A, additional jets are generated by the parton shower). The latter process can lead to events being classified as signal when light jets are misidentified as *b*-jets or when a gluon splits into a $b\bar{b}$ pair during the parton shower. Even with a small light-jet mistag rate of ${\mathscr {O}}(1\%)$, it can have a contribution to the total background comparable to or bigger than the 4*b* process. Details of the generation of the QCD backgrounds and on the associated validation tests are presented in Appendix A and Appendix C.

**Table 4:** Same as Table 3, now listing separately each background process

	Acceptance	VBF	Higgs reco.	$m_{hh}$ cut
LHC 14 TeV				
4*b*	$1.18\times 10^{4}$	613	54	4.45
2*b*2*j*	$1.14\times 10^{5}$	$4.31\times 10^{3}$	514	42.6
$t\bar{t}jj$	150	4.75	0.732	0.0706
$gg\rightarrow hh$	0.98	0.0388	0.0223	0.00857
Total	$1.3\times 10^{5}$	$4.9\times 10^{3}$	569	47
FCC 100 TeV				
4*b*	$3.93\times 10^{5}$	$4.59\times 10^{4}$	$2.61\times 10^{3}$	106
2*b*2*j*	$1.52\times 10^{6}$	$1.46\times 10^{5}$	$6.88\times 10^{3}$	104
$t\bar{t}jj$	$9.76\times 10^{3}$	832	55	1.47
$gg\rightarrow hh$	24.8	2.48	1.31	0.0892
Total	$1.9\times 10^{6}$	$1.9\times 10^{5}$	$9.5\times 10^{3}$	212

Concerning gluon-fusion Higgs pair production in association with additional hard jets, similarly to single-Higgs VBF production there will be certain configurations that mimic the VBF topology as emphasized, for example, in Ref. [64]. In contrast to the VBF channel, however, the Higgs pair production in gluon fusion does not exhibit any enhancement in the tail of the $m_{hh}$ distribution. This substantially reduces its contamination to the region with the highest sensitivity to $c_{2V}$ in our analysis. Note also that a harder $m_{hh}$ distribution could be generated by higher-order EFT operators, for instance those leading to a contact interaction of the form *gghh*, as in Ref. [17]. The investigation of this scenario is, however, left for future work.

In Table 4, following the structure of Table 3, we give the cross sections at 14 and 100 TeV for the individual background processes (and their sum) after the acceptance, VBF, Higgs reconstruction, and $m_{hh}$ cuts. We find that in all steps in the cut-flow the dominant background component is QCD multijet production, both at 14 and 100 TeV. After all analysis cuts, the 2*b*2*j* component is a factor 10 larger than 4*b* at 14 TeV while they are of similar size at 100 TeV.

Other backgrounds, including gluon-fusion di-Higgs production, are much smaller than QCD multijets. Note however, that the former is actually larger than the VBF signal for SM couplings with a cross section at 14 TeV of $0.98 \, (0.009)\,$fb after acceptance (all) cuts, compared to $0.11 \, (0.002)\,$fb for the VBF case. On the other hand, this fact does not affect the measurement $c_{2V}$, since, as we show next, the sensitivity comes from the large $m_{hh}$ tail where the gluon-fusion component is heavily suppressed.

**Fig. 10:**
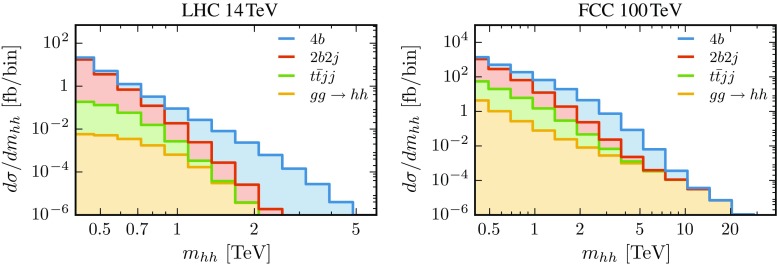
Decomposition of the total background into individual processes as a function the di-Higgs invariant mass after all analysis cuts have been imposed, except for the $m_{hh}$ cut

The decomposition of the total background in terms of individual processes as a function of $m_{hh}$ is shown in Fig. 10 where the components are stacked on top of each other so that the content of each bin matches the total background cross section. In the $14\,$TeV case, the 4*b* background dominates for large $m_{hh}$ while the 2*b*2*j* one is instead the most important for small $m_{hh}$. The $100\,$TeV case is similar with one exception, namely that the gluon-fusion di-Higgs background becomes the dominant one for very high invariant masses, $m_{hh}\gtrsim 10\,$TeV. Such an extreme region, however, is phenomenologically irrelevant due to the very small rates of both signal and background even at a 100 TeV collider.

## Results

The last column of Table 3 indicates the cross sections for the signal and total background after imposing all analysis cuts. We observe that the background, dominated by QCD multijets, still has a much larger cross section than the signal, both in the SM and in the $c_{2V}=0.8$ benchmark scenario. As anticipated, the additional handle which we can now exploit to increase the signal significance is the different behaviour of the $m_{hh}$ distribution for the signal and the background, in particular when $c_{2V}$ departs from its SM value. The latter has a sharp fall-off at large $m_{hh}$ values, while, instead, the signal exhibits a much harder spectrum for $c_{2V} \not = 1$. This cross-section growth implies that, for $|\delta _{c_{2V}}|$ sufficiently large, there will be a crossover value of $m_{hh}$ where the signal overcomes the background.

**Fig. 11:**
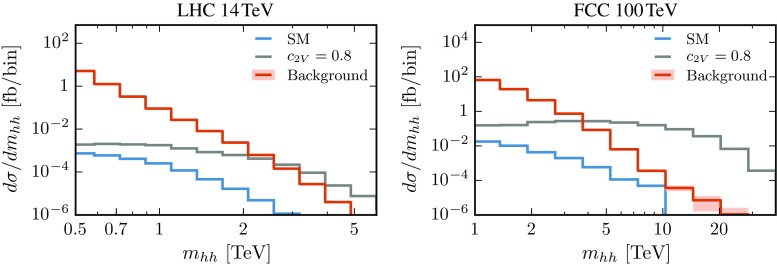
The di-Higgs $m_{hh}$ distribution at 14 TeV (*left*) and 100 TeV (*right*) after all analysis cuts showing the results for the signal (SM and $c_{2V}=0.8$) and for the total background

This behaviour is illustrated in Fig. 11 where we show the invariant mass distribution of the Higgs pairs after all analysis cuts, at 14 and 100 TeV, for the signal (SM and $c_{2V}=0.8$) and the total background. In the case of the benchmark scenario with $c_{2V}=0.8$, the crossover between signal and background is located at $m_{hh}\simeq 2\,$TeV ($4\,$TeV) at $14\,$TeV ($100\,$TeV). We also observe that, for invariant masses $m_{hh}$ above this crossover, the ratio between the signal and the backgrounds keeps increasing steeply.

With the final results of our analysis in hand, we can now estimate the expected sensitivity to deviations in the *hhVV* coupling, parametrized as $\delta _{c_{2V}}=c_{2V}-1$, by exploiting the information contained in the full $m_{hh}$ differential distribution (as opposed to using only the total number of events satisfying all cuts from Table 3). To achieve this, we first bin our results in $m_{hh}$ and then follow a Bayesian approach [109] to construct a posterior probability density function. We include two nuisance parameters, $\theta _B$ and $\theta _S$, to account for the uncertainty associated with the background and signal event rate, respectively. The parameter $\theta _S$ encodes the theoretical uncertainties on the di-Higgs cross section and the branching fraction $\mathrm{BR}(h\rightarrow b\bar{b})$. We conservatively assume a $10\%$ uncertainty uncorrelated in each $m_{hh}$ bin.

**Fig. 12:**
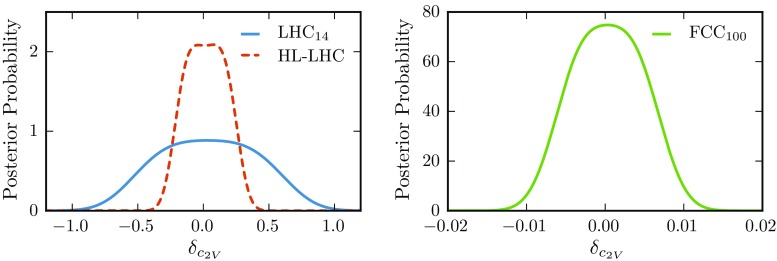
Posterior probability densities for $\delta _{c_{2V}}$ at the LHC for $\mathscr {L}=300$ fb$^{-1}$ (LHC$_{14}$) and $\mathscr {L}=3$ ab$^{-1}$ (HL-LHC) and for the FCC with $\mathscr {L}=10$ ab$^{-1}$. Note the different scales of the axes in the two panels

Concerning $\theta _B$, we expect that an actual experimental analysis of di-Higgs production via VBF would estimate the overall normalization of the different background components by means of data-driven techniques. We assume a 15% uncertainty arising from the measurement and subsequent extrapolation of the dominant QCD multijet background; see for example a recent ATLAS measurement of dijet $b\bar{b}$ cross sections [110]. The background nuisance parameter, $\theta _B$, is conservatively also assumed to be uncorrelated among $m_{hh}$ bins. In addition, while we already rescale the background cross sections to match existing NLO and NNLO results (see Appendix A), there still remains a sizeable uncertainty in their overall normalization from missing higher orders, in particular for the QCD multijet components. For this reason, below, we explore the robustness of our results upon an overall rescaling of all the background cross sections by a fixed factor.

The posterior probability function constructed in this way reads

18 $$\begin{aligned}&P(\delta _{c_{2V}}|\{N^i_\mathrm{obs}\})=\int \! \prod _{i\in \{\text {bins}\}}\! \mathrm{d}\theta _S^i \, \mathrm{d}\theta _B^i\; L\big (N^i(\theta _B^i,\theta _S^i)|N_\text {obs}^i\big ) \nonumber \\&\quad \times \, e^{-(\theta _S^{i})^2/2}\, e^{-(\theta _B^{i})^2/2} \, \pi (c_{2V}), \end{aligned}$$

with $N^i(\theta _B^i,\theta _S^i)$ and $N^i_\text {obs}$ denoting, respectively, the number of predicted (for a generic value of $c_{2V}$) and observed (assuming SM couplings) events for a given integrated luminosity ${\mathscr {L}}$ in the *i*th bin of the di-Higgs invariant mass distribution $m_{hh}$, given by^2^:

19 $$\begin{aligned}&N^i(\theta _B,\theta _S)=\big [ \sigma _\text {sig}^i(c_{2V})\big (1{+}\theta _S^i\,\delta _S\big ){+} \sigma _\text {bkg}^i\big (1{+}\theta _B^i\,\delta _B\big ) \big ] \times {\mathscr {L}}, \nonumber \\&N_\text {obs}^i=\big [ \sigma _\text {sig}^i(c_{2V}=1)+\sigma _\text {bkg}^i \big ] \times {\mathscr {L}}. \end{aligned}$$

In Eq. (19), $\sigma _\text {sig}^i(c_{2V})$ and $\sigma _\text {bkg}^i$ indicate the signal (for a given value of $c_{2V}$) and total background cross sections, respectively, for the *i*-th bin of the $m_{hh}$ distribution. The functional form of $\sigma _\text {sig}^i(c_{2V})$ is given by Eq. (7) and the value of the coefficients in bin *i* are given in Appendix D. We denote by $\pi (c_{2V})$ the prior probability distribution of the $c_{2V}$ coupling.

As justified above, in the evaluation of Eq. (18) we set $\delta _{B(S)} = 0.15\;(0.1)$ and assume that the two nuisance parameters are normally distributed. We have verified that assuming instead a log normal distribution leads to similar results. In addition, we take a Poissonian likelihood $L(N^i|N^i_\text {obs})$ in each bin and assume the prior probability $\pi (c_{2V})$ to be uniform. The resulting posterior probabilities are shown in Fig. 12 for the LHC with $\mathscr {L}=300$ fb$^{-1}$ (LHC$_{14}$) and $\mathscr {L}=3$ ab$^{-1}$ (HL-LHC), and for the FCC with $\mathscr {L}=10$ ab$^{-1}$. To produce this figure, as well as to determine the values reported in Tables 5 and 6, we included all bins with at least one event.

**Table 5:** Expected precision (at 68% probability level) for the measurement of $\delta _{c_{2V}}$ at the LHC and the FCC, assuming SM values of the Higgs couplings. We show results both for the nominal background cross section $\sigma _\text {bkg}$, and for the case in which this value is rescaled by a factor 3

	68% probability interval on $\delta _{c_{2V}}$	
	$1\times \sigma _\text {bkg}$	$3\times \sigma _\text {bkg}$
LHC$_{14}$	[$-0.37$, 0.45]	[$-0.43$, 0.48]
HL-LHC	[$-0.15$, 0.19]	[$-0.18$, 0.20]
FCC$_{100}$	[0, 0.01]	[$-0.01$, 0.01]

**Table 6:** 95% probability upper limits on the fiducial signal strength, $\mu = \sigma /\sigma _\text {sm}$

	95% probability upper limit on $\mu$	
	$1\times \sigma _\text {bkg}$	$3\times \sigma _\text {bkg}$
LHC$_{14}$	109	210
HL-LHC	49	108
FCC$_{100}$	12	23

From Fig. 12, we can determine the expected precision for a measurement of $\delta _{c_{2V}}$ at the LHC and the FCC in the case of SM values of the Higgs couplings. The 68% probability intervals for the determination of $c_{2V}$ at the LHC and the FCC are listed in Table 5. This is the central result of this work. To assess its robustness with respect to our estimate of the background cross sections, we also provide the same intervals in the case of an overall rescaling of the total background by a factor 3. Furthermore, we can also assess the effect of varying $c_V$ on the bound on $\delta _{c_{2V}}$ by treating $c_V$ as a nuisance parameter and marginalizing over it. The leading effect of varying $c_V$ comes from the $(c_{2V}-c_V^2)$ term at the amplitude level – see Eq. (4) – and can be included using the parametrization of Eq. (8). The neglected dependence is subleading and arises from the interference of diagrams proportional to $c_V^2$ and $c_Vc_3$. We take $c_V$ to be Gaussian distributed with a mean equal to 1 (i.e., its SM value) and a width equal to 4.3, 3.3, and 2% at the LHC Run II, HL-LHC, and FCC respectively. In case of the LHC (both Run II and HL), the width of the Gaussian corresponds to the projected sensitivity from the two parameter fit by ATLAS [111]. The effect of marginalizing over $c_V$ is subleading in both LHC scenarios and weakens the bound on $\delta _{c_{2V}}$. We find that the results of Table 5 change by 2% for LHC$_{14}$ and 7% for HL-LHC. The effect at the FCC is much larger causing the bound on $\delta _{c_{2V}}$ to be $\mathscr {O}(0.04)$ rather than 0.01. This is not surprising and indicates that a joint likelihood would be required at the FCC.

**Fig. 13:**
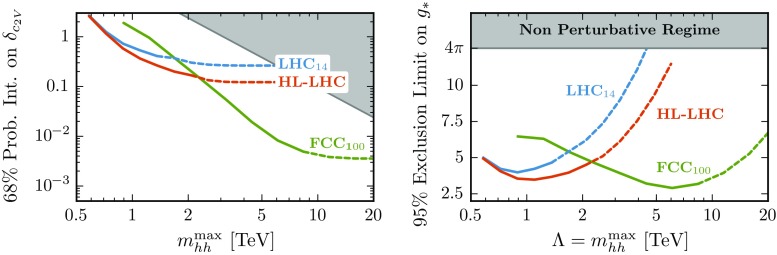
*Left* the expected precision for a measurement of $\delta _{c_{2V}}$ at the 68% CL as a function of $m_{hh}^{\max }$ for the LHC and the FCC. The *grey area* indicates the region where $\delta _{c_{2V}}> \delta _{c_{2V}}^\text {max}$, obtained by setting $\Lambda = m_{hh}^\text {max}$. *Right* the 95% CL exclusion limits in the $(\Lambda =m_{hh}, g_*)$ plane, assuming Eq. (20), where again the *grey area* corresponds to the non-perturbative regime, defined by $g_* \ge 4\pi$. The transition between *solid* and *dashed curves* occurs at the last bin with at least one event

From Table 5 we find that the $c_{2V}$ coupling, for which there are currently no direct experimental constraints, can already be measured at the LHC with $300\,\text {fb}^{-1}$ with a reasonably good accuracy: $_{-37\%}^{+45\%}$ with 68% probability. This accuracy is only marginally degraded if the background is increased by a factor 3. A better precision, of the order of $_{-15\%}^{+19\%}$, is expected at the HL-LHC with $3\,\text {ab}^{-1}$. This estimate is robust against an overall rescaling of the background cross section. Finally, we find a very significant improvement at the FCC with $10\,\text {ab}^{-1}$, where a measurement at the 1% level could be achieved, providing an unprecedented test for our understanding of the Higgs sector.

It is interesting to compare these results with the experimental precision expected on the fiducial VBF di-Higgs production cross section after all analysis cuts, expressed in terms of the signal strength parameter normalized to the SM result, $\mu = \sigma /\sigma _\text {sm}$. Table 6 shows the 95% upper limits on $\mu$ for the nominal background cross section and after rescaling the latter by a factor 3. The comparison with Table 5 clearly shows that the high precision expected on $c_{2V}$ can obtained despite the rather loose constraints that can be obtained on the VBF di-Higgs cross section even at $100\,$TeV. As already discussed, this behaviour follows from the strong dependence of the signal cross section on $c_{2V}$; see Fig. 9.

The results of Tables 5 and 6 have been obtained by making full use of the information contained on the di-Higgs invariant mass distribution $m_{hh}$. However, the EFT expansion might break down at large enough values of $m_{hh}$, corresponding to large partonic centre-of-mass energies, and some assessment on the validity of our procedure is thus required. In particular, results can be consistently derived within the EFT framework only if the new physics scale $\Lambda$ is smaller than the largest value of $m_{hh}$ included in the analysis.

As stressed in Ref. [97], constraining $\Lambda$ requires making assumptions on the structure of the UV dynamics extending the SM. For example, for the case where the new physics is characterized by a single coupling strength $g_*$ and mass scale $\Lambda$ [3], one naively expects

20 $$\begin{aligned} \delta _{c_{2V}}\approx g_*^2 v^2/\Lambda ^2. \end{aligned}$$

Therefore, for maximally strongly coupled UV completions (with $g_* \simeq 4\pi$) it is possible to derive the following upper limit,

21 $$\begin{aligned} \delta _{c_{2V}}^\text {max} \approx 16\pi ^2 v^2/\Lambda ^2, \end{aligned}$$

which makes explicit the connection between the value of $\delta _{c_{2V}}$ and the new physics scale $\Lambda$. The validity of the EFT can thus be monitored by introducing a restriction on the $m_{hh}$ bins used in the construction of the posterior probability Eq. (18), so that $m_{hh} \le m_{hh}^\text {max}$, and then determining how the sensitivity on $\delta _{c_{2V}}$ varies as a function of $m_{hh}^\text {max}$ [97].

The precision on ${\delta _{c_{2V}}}$, defined though the symmetrized 68% probability interval, is shown in Fig. 13 as a function of $m_{hh}^{\max }$ for the LHC and the FCC. As expected, increasing $m_{hh}^\text {max}$, i.e. making the cut less stringent, leads to stronger constraints. Eventually, $\delta _{c_{2V}}$ flattens out when $m_{hh}^\text {max}$ is large enough that all the $m_{hh}$ bins which contain at least one event are included in the posterior probability of Eq. (18). Bins which contain at least one event are depicted in Fig. 13 with a solid curve, while those containing less than one event are depicted by a dashed curve. The grey area in Fig. 13 corresponds to the non-perturbative region where $\delta _{c_{2V}}> \delta _{c_{2V}}^\text {max}$, obtained by setting $\Lambda = m_{hh}^\text {max}$ in Eq. (21), the most optimistic assumption compatible with the validity of the EFT expansion.

As an additional way to quantify the validity of the EFT approach in our analysis, we derive the region of exclusion in the plane $(\Lambda , g_*)$ [97], corresponding to the limits on $\delta _{c_{2V}}$ derived as a function of $m_{hh}^\mathrm{max}$. This is shown in the left panel of Fig. 13, making use of Eq. (20) and then setting $\Lambda = m_{hh}^\text {max}$. The grey area in the upper part of the plot indicates the non-perturbative region, defined by $g_*\ge 4\pi$. We find that the dominant constraints on $\delta _{c_{2V}}$ arise from a region in the parameter space where the EFT expansion is valid, both at the LHC and at the FCC. The results from the two panels of Fig. 13 indicate that our EFT analysis is robust and that it can be used to derive stringent bounds on $\delta _{c_{2V}}$ in the absence of new explicit degrees of freedom.

**Fig. 14:**
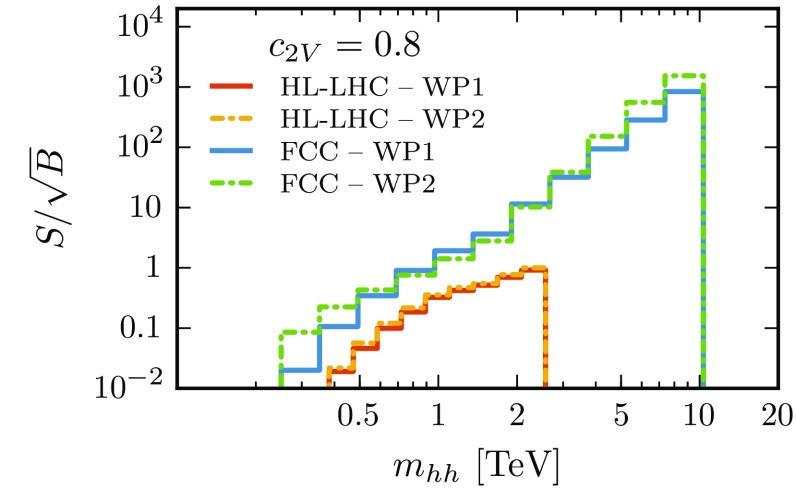
Signal significance $S/\sqrt{B}$ in the $c_{2V}=0.8$ scenario, as a function of the di-Higgs invariant mass $m_{hh}$ at the HL-LHC and the FCC, for the two *b*-tagging working points of Eq. (13)

Finally, in Fig. 14 we show the signal significance, $S/\sqrt{B}$, in the $c_{2V}~$= 0.8 scenario as a function of the di-Higgs invariant mass $m_{hh}$ at the HL-LHC and the FCC. The results are presented for the two *b*-tagging working points defined in Eq. (13). As already discussed, these have been chosen so that the first (WP1) is consistent with the current ATLAS and CMS performances, while the second (WP2) assumes an improved detector performance. One can observe that the signal significance of each individual bin is at most $S/\sqrt{B}\simeq 2$ at the HL-LHC (though the precise numbers depend on the specific choice of binning), while at the FCC one finds much higher signal significances, with $S/\sqrt{B}\simeq 5$ already for $m_{hh}\simeq 1.5$ TeV and then increasing very rapidly for higher values of $m_{hh}$. Figure 14 clearly shows that the signal significance depends very mildly on the specific details of the *b*-tagging performance and that operating at WP2 instead of WP1 implies only a minor improvement.

To summarize, we have demonstrated how Higgs boson pair production via VBF can be used to provide the first direct constraints on the $c_{2V}$ coupling already at the LHC with $\mathscr {L}=300$ fb$^{-1}$ (Table 5), which at a $100\,$TeV collider would become a high-precision measurement with potentially sub-percent accuracy. We have also assessed (Fig. 13) the robustness of our strategy and the validity of the underlying EFT expansion. Our analysis clearly highlights the unique physics potential of extending current di-Higgs searches at the LHC to the vector-boson fusion channel.

## Conclusions and outlook

The measurement and study of Higgs pair production is one of the cornerstones of the LHC program as well as of any future hadron collider. It provides unique information on the Higgs sector and on the mechanism underlying electroweak symmetry breaking, and allows a direct test of the strength of the Higgs boson self-interactions. On the other hand, it is a challenging measurement and the low production rates require large integrated luminosities to achieve reasonable signal significances. While most studies of Higgs pair production so far have concentrated on the gluon-fusion channel which has the largest cross section, we have shown in this work how the vector-boson fusion channel can impose stringent constraints on Higgs couplings that are not directly accessible by other means, in particular on the *hhVV* quartic coupling $c_{2V}$.

Exploiting the high signal yield of the $b\bar{b}b\bar{b}$ final state, we have presented a detailed feasibility study of the measurement of Higgs boson pairs in the vector-boson fusion channel at the LHC and at a future 100 TeV hadron collider. A key ingredient of our strategy is provided by the fact that deviations of the Higgs couplings to vector bosons from the parabola $c_{2V}=c_V^2$ significantly harden the $m_{hh}$ distribution resulting in a large fraction of events with a boosted Higgs pair. The subsequent decays into $b\bar{b}$ pairs can then be reconstructed by means of jet substructure techniques. While QCD backgrounds are very large, we have shown how the combination of selection cuts exploiting the VBF topology and the growth of the $m_{hh}$ distribution when the Higgs couplings depart from their SM values leads to a remarkable model-independent sensitivity to the $c_{2V}$ coupling.

Our results demonstrate that at the LHC with an integrated luminosity of $\mathscr {L}=300$ (3000) fb$^{-1}$ the *hhVV* coupling can be measured with $_{-37\%}^{+45\%}$ ($_{-15\%}^{+19\%}$) precision at the 68% probability level, reaching 1% accuracy at a $100\,$TeV collider. Therefore, stringent constraints on this so far unknown coupling can be obtained already before the start of the HL-LHC data taking. Our analysis provides strong motivation for the ATLAS and CMS collaborations to extend their searches for Higgs pair production to the VBF channel already during Runs II and III. On the other hand, we also find that the VBF channel is clearly inferior to the gluon-fusion channel for a measurement of the Higgs self-coupling $\lambda$, at least for the $b\bar{b}b\bar{b}$ final state studied here, since the sensitivity to $\lambda$ arises from the threshold region $m_{hh}\simeq 2m_h$ where QCD backgrounds overwhelm the signal even for sizeable modifications of the Higgs couplings with respect to their SM values.

There are several possible avenues for future work. On one hand, it might be interesting to study the possibility to enhance the sensitivity to $c_{2V}$ by means of a multivariate analysis (MVA), such as those used in [43], in order to dynamically determine the optimal set of selection cuts and optimize the discrimination between signal and background events. Further, it should be possible to quantify the constraints on additional EFT operators that can contribute to the di-Higgs VBF signal yield and that have not been considered in this work. Finally, a complete analysis should include a full detector simulation, especially for the reconstruction of the forward jets and of the Higgs boson candidates, and a *b*-tagging strategy able to reproduce more closely the one adopted by the LHC experiments.

## Figures and Tables

**Fig. 15:**
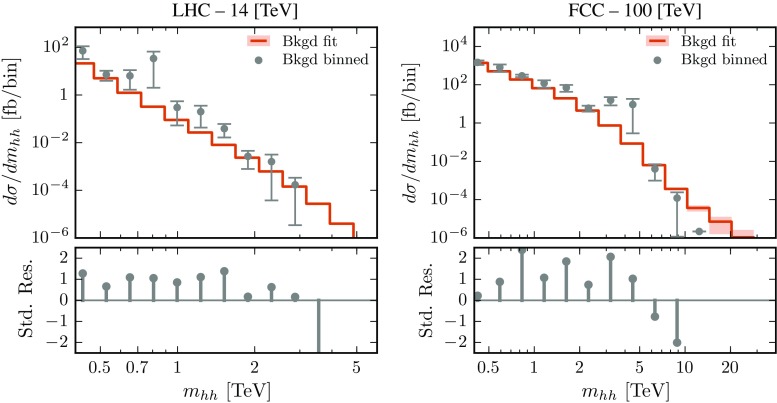
Invariant mass distribution of the di-Higgs system for the total background at 14 TeV (*left*) and 100 TeV (*right*). The histograms are obtained by summing the contributions from the separate fits to each background processes. We also show the binned MC events (*filled circles*) with their statistical uncertainty. The *lower panels* show the standardized residuals in each bin

**Fig. 16:**
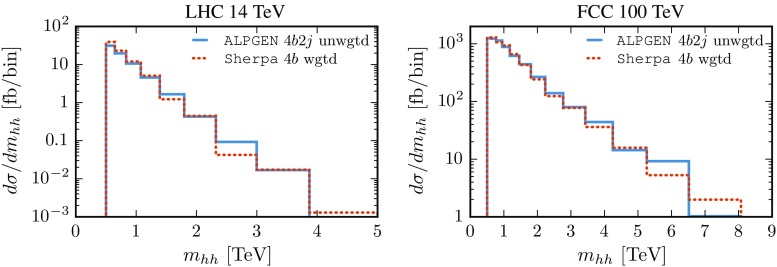
Di-Higgs invariant mass distribution for the 4*b* background, comparing the results of ALPGEN, where unweighted events are generated with 4*b*2*j* matrix elements, with those of SHERPA, where weighted events are generated with 4*b* matrix elements, at 14 TeV (*left*) and 100 TeV (*right*)

**Fig. 17:**
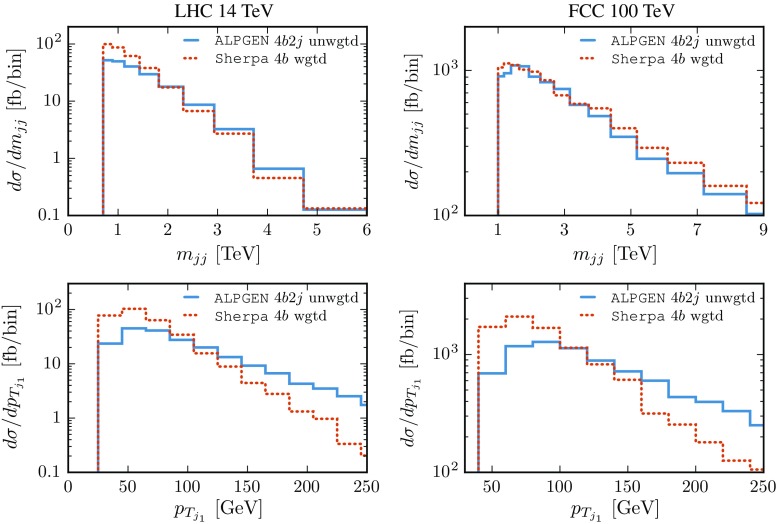
Same as Fig. 16, now for the invariant mass of the two VBF tagging jets $m_{jj}$ (*upper plots*) and the transverse momentum of the hardest light jet in the event $p_{Tj_1}$ (*upper plots*)

## Appendix A: Monte Carlo event generation

In this appendix we discuss the event generation of the signal and background process used in this analysis. In each case, we discuss the programs used, the input parameters and theoretical settings, and the cross-checks that have been performed.

Signal events for Higgs pair production via VBF have been generated at leading order (LO) with MadGraph5 aMC@NLO [112] using a tailored UFO [113] model that allows for generic values of the $c_V$, $c_{2V}$, and $c_3$ couplings in the Lagrangian Eq. (2) (see [114, 115] for other UFO models of Higgs EFTs). We generated 1M unweighted events for each value of $c_{2V}$ and for centre-of-mass energies of 14 and 100 TeV. The size of the signal sample is dictated by the condition of achieving an adequate coverage of the large $m_{hh}$ region. Even so, for small deviations of $c_{2V}$ with respect to its SM value, this region is very difficult to populate. We overcome this limitation by performing a fit to the cross section in each $m_{hh}$ bin as a function of $c_{2V}$ using the general parametrization of Eq. (7).

The MadGraph5_aMC@NLO generation of signal events uses the NNPDF2.3LO [116] set with $\alpha _S(m_Z)=0.119$ interfaced via LHAPDF6 [117]. The calculation is performed in the $n_f=4$ scheme and the factorization and renormalization scales are taken to be the *W* boson mass, $\mu _F=\mu _R=M_W$. To account for higher-order effects, we apply an NNLO/LO *K*-factor ${\simeq } 1.1$ on the inclusive cross section [63] both at 14 and 100 TeV. The decays of the Higgs bosons into $b\bar{b}$ pairs are performed within MadGraph5_aMC@NLO with adjusted parameters to ensure that the branching ratio corresponds to Higgs Cross-Section Working Group value [35] of $\mathrm{BR}(h\rightarrow b\bar{b})=0.582$. Since in this work we only modify the $c_{2V}$ coupling, assuming the SM value of the total Higgs width is a very good approximation.

With this setup, we have generated signal events in the SM and for a wide range of values of $\delta _{c_{2V}}$. At the matrix-element level, we applied the generation cuts listed in Table 7. The corresponding cross sections are summarized in Table 8, where we provide the SM results as well as the predictions for various BSM scenarios defined by the couplings $\{c_V,c_{2V},c_3\}$, including the benchmark scenario $c_{2V}=0.8$. In addition, the number of expected events at the HL-LHC, assuming an integrated luminosity of $\mathscr {L}=3\,\mathrm{ab}^{-1}$, and at the FCC 100 TeV for $\mathscr {L}=10\,\mathrm{ab}^{-1}$, are also listed.

**Table 7:** Parton-level generation cuts for the VBF di-Higgs signal and the QCD background samples at $\sqrt{s}=14$ and 100 TeV. In this table, *j* refers to light quarks and gluons, *b* to bottom quarks, and $m_{jj}$ is the invariant mass of the two light partons in the event with largest $p_T^j$

	14 TeV		100 TeV	
	Signal	QCD bkg.	Signal	QCD bkg.
$p_{T_j}~\text {(GeV)}~\ge ~$	25	20	25	20
$p_{T_b}~\text {(GeV)}~\ge ~$	25	20	25	20
$|\eta _j|\le ~$	4.5	3	10	3
$|\eta _b|\le ~$	2.5	3	3	3
$\Delta R_{bb}\ge ~$	0.1	0.1	0.1	0.1
$\Delta R_{jb}\ge ~$	0.4	0.1	0.4	0.1
$\Delta R_{jj}\ge ~$	4	0.1	4	0.1
$m_{jj}~\text {(GeV)}~\ge ~$	600	–	600	–

**Table 8:** Parton-level cross sections for VBF Higgs pair production in the $b\bar{b}b\bar{b}$ final state at 14 and 100 TeV (including branching ratios) after the acceptance cuts of Table 7.We show the cross sections for different values of the couplings $\{c_V,c_{2V},c_3\}$ normalized to the SM values. We also provide the number of events at the HL-LHC for $\mathscr {L}=3\,\mathrm{ab}^{-1}$ and at the FCC for $\mathscr {L}=10\,\mathrm{ab}^{-1}$

		Signal: VBF $hh\rightarrow b\bar{b} b\bar{b}$			
		LHC 14 TeV		FCC 100 TeV	
$\{c_V,c_{2V},c_3\}$		$\sigma$ (fb)	$N_\mathrm{ev}\,(\mathscr {L}=3\,\mathrm{ab}^{-1})$	$\sigma$ (fb)	$N_\mathrm{ev}\,(\mathscr {L}=10\,\mathrm{ab}^{-1})$
{1,1,1}	SM	0.26	780	15	$1.5\times 10^5$
{1,0,1}		4.4	$1.3\times 10^4$	593	$5.9\times 10^6$
{1,2,1}		2.5	$7.5\times 10^3$	471	$4.7\times 10^6$
{1,0,0}		5.8	$1.7\times 10^4$	656	$6.6\times 10^6$
{1,0,-1}		7.5	$2.3\times 10^4$	731	$7.3\times 10^6$
{1,1,0}		0.64	$1.9\times 10^3$	30	$3.0\times 10^5$
{1,0.8,1}	Benchmark	0.58	1740	48	$4.8\times 10^5$

Following the parton-level event generation, the resulting Les Houches event files for signal events are showered using the Pythia8 Monte Carlo generator [118] v8.212 using the Monash 2013 Tune [119]. No hadronization or underlying event (UE) effects are included for simplicity. Although we have not attempted to simulate the effects of pile-up (PU) in our analysis, recent studies [43] indicate that modern PU mitigation techniques [120] should be efficient enough to minimize the PU contamination even for the $b\bar{b}b\bar{b}$ final state.

Concerning the generation of the QCD background processes, their parton-level cross sections are summarized in Table 9. In each case we indicate the programs used for the event generation, the number of MC events $N_\mathrm{ev}$ that have been generated, whether the events are weighted or unweighted, and the LO cross sections along with the corresponding higher-order *K*-factors at 14 and 100 TeV. As discussed in Sect. 4, our results include a 15% systematic uncertainty on the total background normalization (assuming a data-driven determination in an experimental analysis), and we also assessed the robustness of our estimate for the measurement of $\delta _{c_{2V}}$ in case of an overall upwards shift of the backgrounds by a factor 3.

For QCD multijet production we have explored two complementary approaches for event generation. First of all, we used ALPGEN [121] to generate a large sample of unweighted events at the matrix-element level and showered them with Pythia8. This approach, however, presents a difficulty, because the differential cross section with respect to the di-Higgs invariant mass $m_{hh}$ falls very rapidly for increasing $m_{hh}$. We thus find that an unrealistically large sample of unweighted events would be needed in order to adequately populate the tail of the $m_{hh}$ distribution.

**Table 9:** Parton-level cross sections for the background processes considered in this work after the acceptance cuts of Table 7. We indicate the programs used, the numbers of events generated $N_\mathrm{ev}$ and whether they are weighted [W] or unweighted [UW], the LO cross sections and the corresponding *K*-factors and the relevant reference. In the case of the 4*b*2*j* and 2*b*4*j* backgrounds, the same *K*-factors as those for the 4*b* and 2*b*2*j*, respectively, were applied. For the 4*b*2*j* sample, the 6M(2M) events correspond to those generated at the LHC(FCC) centre-of-mass energies, respectively. The generation cuts used for the ALPGEN  samples are different; see text

Background processes							
			$\sigma _\mathrm{LO}$ (fb)		*K*-factor		Refs.
Process	Program	$N_\mathrm{ev}$	LHC14	FCC100	LHC14	FCC100	
4*b*	Sherpa2.2	50M [W]	$1.1\times 10^6$	$1.6\times 10^7$	1.7	1.7	[112]
2*b*2*j*	Sherpa2.2	50M [W]	$2.6\times 10^8$	$3.8\times 10^9$	1.3	1.3	[112]
$t\bar{t}jj$	Sherpa2.2	10M [W]	$1.9\times 10^4$	$1.6\times 10^6$	1.6	1.6	[122]
4*b*2*j*	ALPGEN	6M(2M) [UW]	$5.4\times 10^{4}$	$2.4\times 10^{6}$	1.7	1.7	–
2*b*4*j*	ALPGEN	260k [UW]	$10^{7}$	$5.2\times 10^{8}$	1.3	1.3	–
$gg\rightarrow hh\rightarrow b\bar{b} b\bar{b}$	aMC@NLO	1M [UW]	6.2	272	2.4	2.2	[47]

To bypass this limitation, we generated 50M weighted 4*b* and 2*b*2*j* events with Sherpa v2.2 [123] and then processed them with the built-in shower. The main advantage of this approach is that events with small weights are not discarded by the unweighting and, therefore, enough events with large $m_{hh}$ (and thus small weight) are still kept. One possible drawback is that, for the same number of events, the unweighted sample provides a better estimate of the total cross section than the weighted one. In our case this is not an issue, since with our weighted sample we achieve statistical uncertainties of order $2\%$ (to be compared with ${\gtrsim } 0.01\%$ for a same-size unweighted sample), which is more than sufficient for our purposes. On the other hand, to generate a large enough number of events in a reasonable amount of CPU time we are forced to use a lower multiplicity final state, and thus we rely on the parton shower to generate the additional light partons (see Table 9). To validate this procedure, we explicitly verified that, in the region where the two approaches lead to sufficient statistics, the Sherpa calculation based on 4*b* (2*b*2*j*) matrix elements and the ALPGEN one, based on 4*b*2*j* (2*b*4*j*) matrix elements, lead to comparable results (see Appendix C). This agreement indicates that the parton shower does a reasonable job in modelling additional hard radiation in the phase-space region of interest.

The Sherpa samples in Table 9 have been generated using the NNPDF3.0 NNLO set [124] with strong coupling $\alpha _S(m_Z^2)=0.118$ and with $n_f = 4$ active quark flavours, and use as factorization and renormalization scales $\mu _F=\mu _R=H_T/2$, with

A.1 $$\begin{aligned} H_T \equiv \sum _{i}^{n}\sqrt{( m_{t,i})^2+( {p_{T,i}^t})^2}, \end{aligned}$$

where *n* is the final-state matrix-element multiplicity and $m_{t,i}$ and $p_{T,i}^t$ are the transverse mass and transverse momentum of the *i*-th final-state parton. The generation-level acceptance cuts applied to these samples are listed in Table 7. As for the other background samples, LO cross sections have been rescaled by the best available higher-order *K*-factors.

Higgs pair production via gluon fusion is simulated at LO using MadGraph5_aMC@NLO for loop-induced processes [125]. The cross section is rescaled to match the inclusive NNLO+NNLL calculation [47] yielding a *K*-factor, $\sigma _\mathrm{NNLO+NNLL}/\sigma _\mathrm{LO}=2.4~(2.2)$ at $14\,$TeV ($100\,$TeV). Parton-level events are then showered with Pythia8 using the same settings as for the signal VBF di-Higgs samples. No generation cuts are applied, and the resulting cross sections (including the branching fraction into $b\bar{b}b\bar{b}$) are listed in Table 9. While the hard-scattering process that is generated, $gg\rightarrow hh$, does not include additional jets, initial state radiation from the gluon legs can give a VBF-like topology with two forward jets characterized by a large invariant mass, and thus contribute to the total *hhjj* yield. We have verified that our calculation provides a reasonable description of the relevant kinematical distributions, such as $m_{hh}$, by comparing with the $gg\rightarrow hhjj$ process computed in the EFT approximation. As shown in Sect. 4, while the gluon-fusion contamination to the VBF signal is substantial close to threshold, it is marginal in the large $m_{hh}$ region where the sensitivity to $c_{2V}$ is the highest.

## Appendix B: Fitting the tail of the $m_{hh}$ distribution for background processes

The background processes considered in this work (see Appendix A) exhibit a steep fall-off for large values of $m_{hh}$, the invariant mass distribution of the reconstructed Higgs pair. Even with the use of weighted events, it is difficult to adequately populate this region. To obtain a reliable estimate of the cross section there, it is thus necessary to introduce a fitting procedure. In this appendix we discuss how the fits to the $m_{hh}$ distributions for the background processes, and the associated validation tests, have been performed.

We found that, far from the di-Higgs production threshold, the following functional form provides a reasonable description of the $m_{hh}$ distribution:

B.1 $$\begin{aligned} \sigma ^\mathrm{(fit)}(m_{hh},\sqrt{s}) {=} \left[ 1{-}\left( \frac{m_{hh}}{\sqrt{s}}\right) ^{1/4}\right] ^a\left( \frac{m_{hh}}{\sqrt{s}}\right) ^{b+c\cdot \log (m_{hh}/\sqrt{s})}, \end{aligned}$$

where $\sqrt{s}$ is the collider centre-of-mass energy. This specific functional form is a modified version of one of those suggested in Ref. [126] for the fit of the di-photon invariant mass distribution at the LHC. We explored other choices, finding a comparable fit quality.

In Eq. (B.1), the fit parameters $\{a,b,c\}$ are determined by minimizing the $\chi ^2$,

B.2 $$\begin{aligned} \chi ^2 = \sum _{i=1}^n \frac{\big ( \sigma ^\mathrm{(th)}_i-\sigma ^\mathrm{(fit)}(m_{hh}^{(i)},\sqrt{s})\big )^2}{\delta _i^2}, \end{aligned}$$

where *n* is the number of bins in the $m_{hh}$ distribution used as input to the fit, $\sigma ^\mathrm{(th)}_i$ is the theoretical prediction for the cross section in the bin $m_{hh}^{(i)}$, and

B.3 $$\begin{aligned} \delta _i \equiv \sqrt{\sum _{k=1}^{N_i}\big ( w_k^{(i)}\big )^2} \end{aligned}$$

is the statistical uncertainty associated with the $N_i$ weighted Monte Carlo events that populate bin *i* with weights $\{w_k^{(i)}\}$. Note that when the MC events are unweighted, $\delta _i\propto \sqrt{N_i}$, as expected. The resulting fit coefficients are shown in Table 10 for both centre-of-mass energies and for the four background processes considered. We have also verified that the results for the fit parameters are stable with respect to variations of the binning in $m_{hh}$.

**Table 10:** The fit coefficients in Eq. (B.1) for each background process at 14 and 100 TeV

$\sqrt{s}$ (TeV)	Background	*a*	*b*	*c*
14	4*b*	0.492	14.2	0.512
	2*b*2*j*	$-2.65$	24.9	0.198
	*ttjj*	$-18.3$	48	$-3.63$
	*ggf*	$-8.27$	30.1	$-1.82$
100	4*b*	$-4.82$	32.3	$-0.539$
	2*b*2*j*	$-2.97$	36.8	$-0.118$
	*ttjj*	$-3.31$	38.4	$-0.265$
	$gg\rightarrow hh$	3.61	15.9	0.695

To validate the fitting procedure, we show in Fig. 15 the $m_{hh}$ distribution of the reconstructed di-Higgs system for the total background, where each individual process has been fitted separately and then added up to construct the solid histograms. The fit results are compared to the cross sections from the Monte Carlo generation (indicated by filled circles) with their corresponding statistical uncertainty, Eq. (B.3). In the lower panels of Fig. 15 we show the fit residuals, defined as the difference between MC and fit divided by the statistical uncertainty in each bin. The fact that the parametrized fit agrees with the MC calculations at the 2-$\sigma$ level or better (in units of the uncertainty of the latter) for a wide range of $m_{hh}$ demonstrate the goodness of these fits.

Note that, in the case of the QCD multijet backgrounds, we exclude the first bin to ensure that the fit is not affected by artificial features in the $m_{hh}$ distribution induced by the analysis selection cuts. Furthermore, in the case of the gluon-fusion di-Higgs background, it is necessary to exclude the first few bins of the $m_{hh}$ distribution from the fit. The reason is that in this case there is a production threshold at $2m_{h}$, and, as a result, the distribution does not decrease monotonically with $m_{hh}$ unless one is far enough from threshold. By excluding these bins, we avoid biasing the resulting fit in the tail of the $m_{hh}$ distribution, the region where a functional form such as Eq. (B.1) does provide an equally satisfactory description as for the rest of background processes.

## Appendix C: Validation of the QCD multijet generation

As discussed in Appendix A, the modelling of the tail of the $m_{hh}$ distribution for background processes is particularly challenging. One reason is because all the backgrounds considered are characterized by a steep power-like fall-off with $m_{hh}$ above the di-Higgs production threshold, and therefore it becomes necessary to introduce a cross-section parametrization (see Appendix B) to be able to cover this region.

In addition, in the case of the QCD multijet backgrounds, there are different options available for the modelling of the $m_{hh}$ distribution, in particular the multiplicity of the matrix-element calculation prior to the parton shower. Ideally, one should generate all relevant final-state partonic multiplicities and merge them to avoid double counting, either at LO [127–129] or at NLO [130, 131]. For the purposes of this feasibility study, however, we found it sufficient to generate LO samples using 4*b* and 2*b*2*j* matrix elements with Sherpa, with additional hard radiation provided by the parton shower. Note that our approach could introduce some double counting from gluon splittings into $b\bar{b}$, which if anything would increase the background cross section and make our estimates of $\delta _{c_{2V}}$ more conservative. Moreover, let us recall that, as discussed in Sect. 4, an actual experimental analysis would estimate the overall background normalization by means of data-driven techniques.

To validate the robustness of our simulation of the $m_{hh}$ distributions for the QCD multijet background, we have compared the Sherpa calculation, based on 4*b* and 2*b*2*j* matrix elements and weighted events, with the ALPGEN simulation, based on 4*b*2*j* and 2*b*4*j* matrix elements and unweighted events showered with Pythia8. In principle, ALPGEN should provide a better description of the kinematics of the VBF jets, since it is based on higher-multiplicity matrix elements but it has the drawback that populating the tail of the $m_{hh}$ with unweighted events is very CPU-time intensive. On the other hand, it turns out that the two approaches give comparable results for the $m_{hh}$ distribution in the region where both approaches lead to sufficient MC statistics, validating the use of the Sherpa for the calculation of background cross sections.

In Fig. 16 we show the $m_{hh}$ distribution for the 4*b* background at 14 and 100 TeV, comparing the ALPGEN and SHERPA calculations. We find good agreement for the entire $m_{hh}$ range, indicating that the SHERPA event generation does a reasonable job in modelling the VBF tagging jets, and demonstrating that it can be reliably used to populate the large $m_{hh}$ region for the multijet backgrounds with high efficiency. The differences between the two calculations are at most a factor two, typically less, well within the typical size of the theoretical uncertainties for LO multijet calculations.

Figure 17 shows a similar comparison for the invariant mass of the two VBF tagging jets, $m_{jj}$, and for the transverse momentum of the hardest light jet in the event, $p_{Tj_1}$. For the $m_{jj}$ distribution, the agreement between the ALPGEN and SHERPA calculations is also good for the entire kinematical range. On the other hand, for the transverse momentum of the leading jet the ALPGEN calculation leads to harder (softer) spectra than the SHERPA one at high (low) values of $p_{Tj_1}$ . This can be understood from the fact that $j_1$ will be typically generated by the parton shower in the latter case, and by the matrix element in the former. These differences are however inconsequential for our analysis, since the cuts imposed on the $p_T$ of the VBF tagging jets are relatively mild, see Table 2, and thus the event selection will not be affected.

The validation studies discussed in this appendix demonstrate that, for the purposes of the present analysis, our approach to event generation based on SHERPA for the simulation of the QCD multijet backgrounds is justified. On the other hand, they also highlight that future studies aiming to enhance the separation between signal and background events from shape comparisons of kinematical distributions, such as multivariate analysis [43], would require an improved modelling of the QCD multijet backgrounds. This could be achieved by using merging techniques to combine QCD jet samples of different multiplicities.

## Appendix D: Coefficients of the $\delta _{c_{2V}}$ fit

The dependence of the signal cross section on $\delta _{c_{2V}}$, as parametrized in Eq. (7), is required in order to construct the likelihood function. In this appendix, we list the coefficients of Eq. (7) in each $m_{hh}$ bin. The coefficients are extracted by fitting MC events after all cuts have been applied as discussed in Sect. 3 with the exception of the $m_{hh}$ cut. We use 15 equally spaced bins on a log scale starting from 250 GeV up to 6(30) TeV for $\sqrt{s}=14(100)$ TeV. In addition, we define an overflow bin up to the centre-of-mass energy. The results are shown in Tables 11 and 12. The fit error on each coefficient is also provided along with the off-diagonal entries of the correlation matrix $\rho _{ij}$ where $i,j\in \{\sigma ,A,B\}$.

**Table 11:** The bin by bin fit coefficients obtained by fitting MC events to Eq. (7) for the LHC with $\sqrt{s}=14$ TeV. The first column labelled ‘Bin’ gives the bin number in question. The bin definition is given in the text; see also footnote 2. The last three columns labelled $\rho _{0A}$, $\rho _{0B}$, and $\rho _{AB}$ give the coefficients of the correlation matrix among the three fit parameters

Bin	$\sigma _\text {sm}$ (fb)	*A*	*B*	$\rho _{\sigma A}$	$\rho _{\sigma B}$	$\rho _{AB}$
1	$(3.08\pm 0.05)\times 10^{-4}$	$-3.61 \pm 0.0957$	$6.89 \pm 0.217$	0.3	$-0.62$	$-0.66$
2	$(5.84\pm 0.0691)\times 10^{-4}$	$-3.96 \pm 0.0706$	$7.27 \pm 0.161$	0.32	$-0.62$	$-0.68$
3	$(7.17\pm 0.0654)\times 10^{-4}$	$-4.3 \pm 0.0573$	$9.71 \pm 0.148$	0.32	$-0.68$	$-0.63$
4	$(7.31\pm 0.049)\times 10^{-4}$	$-4.96 \pm 0.0462$	$14.9 \pm 0.146$	0.35	$-0.77$	$-0.59$
5	$(5.98\pm 0.0507)\times 10^{-4}$	$-6.39 \pm 0.0677$	$26.7 \pm 0.287$	0.42	$-0.86$	$-0.59$
6	$(4.19\pm 0.0677)\times 10^{-4}$	$-8.28 \pm 0.157$	$50.1 \pm 0.93$	0.5	$-0.92$	$-0.6$
7	$(2.38\pm 0.0571)\times 10^{-4}$	$-11.9 \pm 0.302$	$103 \pm 2.65$	0.61	$-0.96$	$-0.67$
8	$(1.15\pm 0.0447)\times 10^{-4}$	$-15.1 \pm 0.597$	$174 \pm 7.05$	0.67	$-0.98$	$-0.71$
9	$(4.93\pm 0.176)\times 10^{-5}$	$-19.4 \pm 0.712$	$301 \pm 11$	0.72	$-0.99$	$-0.75$
10	$(1.68\pm 0.148)\times 10^{-5}$	$-29.1 \pm 2.65$	$758 \pm 67.8$	0.8	$-1$	$-0.81$
11	$(5.41\pm 1.02)\times 10^{-6}$	$-42.3 \pm 8.58$	$1.69\times 10^{3} \pm 320$	0.83	$-1$	$-0.83$
12	$(1.28\pm 0.506)\times 10^{-6}$	$-47.4 \pm 22.6$	$(3.88\pm 1.53)\times 10^{3}$	0.76	$-1$	$-0.77$
13	$(8.55\pm 13.4)\times 10^{-7}$	$-16.5 \pm 29.7$	$(2.27\pm 3.57)\times 10^{3}$	0.76	$-1$	$-0.77$
14	$(3.5\pm 3.57)\times 10^{-7}$	$-0.00901 \pm 26.4$	$(1.28\pm 1.31)\times 10^{3}$	$-0.064$	$-1$	0.055
15	$(6.24\pm 3.38)\times 10^{-7}$	$-3.59 \pm 5.3$	$105 \pm 60.7$	0.19	$-0.98$	$-0.24$
16	$(6.33\pm 1.97)\times 10^{-7}$	$-2.88 \pm 1.57$	$22.1 \pm 8.59$	0.15	$-0.9$	$-0.36$

**Table 12:** Same as Table 11 but for the FCC with $\sqrt{s}=100$ TeV

Bin	$\sigma _\text {sm}$ (fb)	*A*	*B*	$\rho _{\sigma A}$	$\rho _{\sigma B}$	$\rho _{AB}$
1	$(5.98\pm 0.152)\times 10^{-3}$	$-2.87 \pm 0.191$	$7.8 \pm 0.513$	0.14	$-0.57$	$-0.35$
2	$(1.43\pm 0.0185)\times 10^{-2}$	$-4.29 \pm 0.106$	$11.8 \pm 0.317$	0.23	$-0.62$	$-0.46$
3	$(2.25\pm 0.0246)\times 10^{-2}$	$-5.63 \pm 0.0999$	$21.9 \pm 0.395$	0.29	$-0.74$	$-0.46$
4	$(2.36\pm 0.045)\times 10^{-2}$	$-7.95 \pm 0.212$	$50.8 \pm 1.27$	0.37	$-0.86$	$-0.47$
5	$(1.88\pm 0.0277)\times 10^{-2}$	$-11.7 \pm 0.203$	$109 \pm 1.86$	0.5	$-0.93$	$-0.56$
6	$(1.14\pm 0.0226)\times 10^{-2}$	$-14.6 \pm 0.339$	$210 \pm 4.5$	0.56	$-0.97$	$-0.6$
7	$(5.68\pm 0.196)\times 10^{-3}$	$-24.9 \pm 0.985$	$729 \pm 25.8$	0.69	$-0.99$	$-0.7$
8	$(2.73\pm 0.224)\times 10^{-3}$	$-43.2 \pm 4.07$	$2.05\times 10^{3} \pm 170$	0.77	$-1$	$-0.78$
9	$(1.1\pm 0.104)\times 10^{-3}$	$-61.3 \pm 7.08$	$5.54\times 10^{3} \pm 523$	0.77	$-1$	$-0.77$
10	$(2.97\pm 0.806)\times 10^{-4}$	$-135 \pm 43.2$	$(1.96\pm 0.532)\times 10^{4}$	0.83	$-1$	$-0.83$
11	$(4.83\pm 2.99)\times 10^{-5}$	$-66.2 \pm 124$	$(9.7\pm 6.01)\times 10^{4}$	0.32	$-1$	$-0.32$
12	$(1.71\pm 1.57)\times 10^{-5}$	$-114 \pm 276$	$(1.88\pm 1.73)\times 10^{5}$	0.37	$-1$	$-0.37$
13	$(1.39\pm 31.2)\times 10^{-5}$	$-163 \pm 3.64\times 10^{3}$	$(1.21\pm 27.3)\times 10^{5}$	1	$-1$	$-1$
14	$(4.32\pm 219)\times 10^{-6}$	$-1.59 \pm 689$	$(1.35\pm 68.1)\times 10^{5}$	$-0.012$	$-1$	0.012
15	$(2.66\pm 6.97)\times 10^{-5}$	$-0.0026 \pm 32.2$	$(3.53\pm 9.29)\times 10^{3}$	$-0.091$	$-1$	0.09
16	$(2.6\pm 1.8)\times 10^{-5}$	$-0.0018 \pm 7.38$	$144 \pm 109$	$-0.38$	$-0.98$	0.36

## Footnotes

1 Note that we start with the six hardest *b*-jets since gluon splitting generates additional *b*-jets. Thus, there is a non-zero probability of missing signal *b*-jets (i.e. from Higgs decays) if we only restrict ourselves to the hardest four.

2 In our analysis, we use 15 bins starting at 250 GeV up to 6(30)TeV for the LHC(FCC) that are uniformly spaced on a log scale. In addition, we define an overflow bin up to the relevant centre-of-mass energy.
